# Artisanal Green Turtle, *Chelonia mydas*, Fishery of Caribbean Nicaragua: I. Catch Rates and Trends, 1991–2011

**DOI:** 10.1371/journal.pone.0094667

**Published:** 2014-04-16

**Authors:** Cynthia J. Lagueux, Cathi L. Campbell, Samantha Strindberg

**Affiliations:** Global Conservation Program, Wildlife Conservation Society, Bronx, New York, United States of America; University of Wales Swansea, United Kingdom

## Abstract

This is the first assessment of catch rates for the legal, artisanal green turtle, *Chelonia mydas*, fishery in Caribbean Nicaragua. Data were collected by community members, monitoring up to 14 landing sites from 1991 to 2011. We examined take levels, and temporal and spatial variability in catch rates for the overall fishery, by region, and community using General Additive Mixed Models (GAMMs). More than 171,556 green turtles were killed during the period, with a mean estimated minimum 8,169±2,182 annually. There was a statistically significant decline in catch rates overall. Catch rates peaked in 1997 and 2002, followed by a downward trend, particularly from mid-2008 to the end of the study period. Similar downward trends were evident in both study regions. Community specific catch rate trends also indicated declines with decreases ranging from 21% to 90%. Decrease in catch rates in Nicaragua is cause for concern even though the principal source rookery at Tortuguero, Costa Rica, shows an increase in nesting activity. Explanations for the apparent discrepancy between the increasing trend at Tortuguero and decreasing catch rate trends in Nicaragua include: i) an increase in reproductive output, ii) insufficient time has passed to observe the impact of the fishery on the rookery due to a time lag, iii) changes in other segments of the population have not been detected since only nesting activity is monitored, iv) the expansive northern Nicaragua foraging ground may provide a refuge for a sufficient portion of the Tortuguero rookery, and/or v) a larger than expected contribution of non-Tortuguero rookeries occurring in Nicaragua turtle fishing areas. Our results highlight the need for close monitoring of rookeries and in-water aggregations in the Caribbean. Where consumptive use still occurs, nations sharing this resource should implement scientifically based limits on exploitation to ensure sustainability and mitigate impacts to regional population diversity.

## Introduction

The abundance of sea turtles in the Caribbean at the onset of European arrival to the New World, as well as subsequent skirmishes to conquer and control the lands and resources of the region have been well described by Dampier [1]; Columbus (cited in Lewis [2]); and Exquemelin [3]. In writing about the masses of green turtles (*Chelonia mydas*) in the vicinity of the Cayman Islands, Exquemelin [3] described how ships that had missed landfall, at times, could set their course to the sound of turtles blowing. Parsons [4] reviewed the exploitation and decline of sea turtles around the world, including the drastic decline of green turtles in the Caribbean. More recently, it has been hypothesized that since the arrival of Europeans green turtles have declined as much as 99% in the Caribbean [5], [6], causing large ecosystem changes to seagrass beds over the ensuing centuries [7].

**Figure 1:**
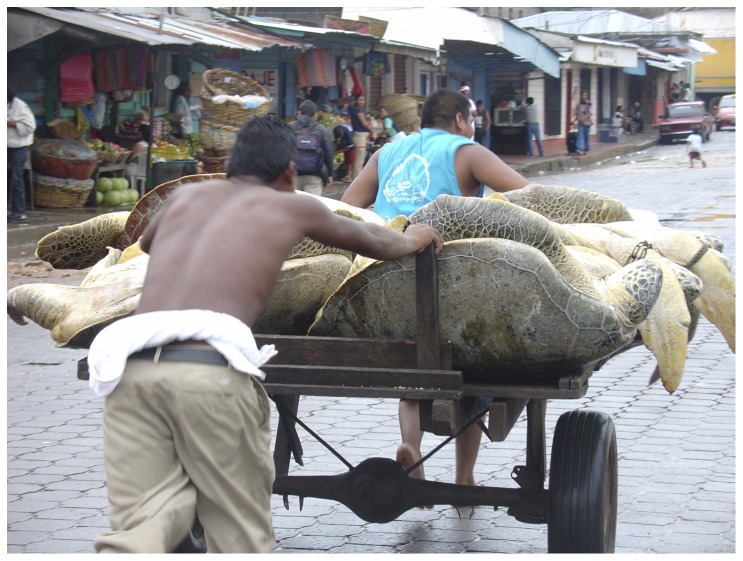
Green turtles, *Chelonia mydas*, being transported to slaughter in Bluefields, RAAS, Nicaragua. Photo credit C.J. Lagueux.

The history of sea turtle exploitation along the Caribbean coast of Nicaragua parallels that of the wider Caribbean. Seagrass beds found on the expansive continental shelf off the Caribbean coast of Nicaragua are thought to be among the largest in the Caribbean, if not the world [8]–[10], and are the principal forage of green turtles [11]. Marine turtles and their eggs have been exploited for at least 500 years from Nicaragua's coastal waters and beaches by indigenous and ethnic coastal inhabitants, and by foreign fleets [1], [4], [12]–[18] (Fig. 1). Despite this long and extensive history of exploitation, Nicaragua's Caribbean coast continues to provide foraging, developmental, and nesting habitats [19], [20], and a reproductive migratory corridor for what has been suggested as the largest green turtle foraging aggregation in the Atlantic [21].

Throughout centuries of resource extraction cycles, the green turtle has been a relatively stable resource contributing towards meeting kinship social obligations through food distribution, and protein and income needs of coastal inhabitants [16], [18], [22]–[25]. As early as 1633, English colonists from Providence Island established a trading station among the Miskitu Indians at Cabo Gracias a Dios, now the Honduras/Nicaragua border [4]. By 1722, Jamaican and possibly Cayman Island boats were annually visiting the Miskito Cays to catch and purchase green turtles, and hawksbill turtle, *Eretmochelys imbricata*, shell from the Miskitu Indians (Fernández cited in Parsons [4]). By the early 1800s, Cayman Islanders were regularly turtling off the coast of Nicaragua once other green turtle stocks were depleted [12]. Simmonds (cited in Parsons [4]) reported that by 1878, up to 15,000 turtles annually were landed in Europe, most of them having been caught by the Cayman turtling fleet, although it is not clear if all were taken from Nicaragua waters.

During the first-half of the 20^th^ century approximately 1,200 to 4,600 green turtles were taken annually [4], [26], and from 1958 to 1967, approximately 1,000 to 2,360 green turtles were exported annually [16] from the Nicaraguan coast by Cayman Island boats. Estimated levels of green turtle take prior to 1967, however, were based on export levels only and did not include take of animals for local consumption. By 1967, the Nicaragua government revoked Cayman Islanders permits to turtle within their waters [16], [27], opting instead to expand their own exploitation of turtles. During the late 1960s and early 1970s, three green turtle packing plants were established on the coast, at Bluefields, Corn Island, and Puerto Cabezas, with financial support from the U.S. Agency for International Development, for the sole purpose of processing green turtles for export [16], [27]–[30]. Between 1968 and 1971, Nietschmann [16] reported between 4,000 and 10,000 green turtles were taken annually from this coast to meet local and international demand. Based only on imports, Cato et al. [29] reported approximately 445,500 kg (equivalent to approximately 10,000 animals) of sea turtle products were imported into the U.S. from Nicaragua during 7 of the 10 years between 1966 and 1976. By 1977, the processing plants were closed [25], [30] and Nicaragua became a signatory to the Convention on International Trade in Endangered Species of Wild Fauna and Flora (CITES) [31]. From 1985 to 1990, a period during the Contra/Sandinista civil war, at least 16,700 (average 2,783/yr) green turtles captured in the Miskito Cays fishing area were landed in Puerto Cabezas and the meat sold in the local market [17].

Regulations to manage the take of green turtles in Nicaragua began in the mid-1900s through the establishment of closed seasons. Nevertheless, enforcement has often been lacking and/or local authorities have relaxed the regulations under pressure of economic hardship by local residents ([16]–[18], [32]; CJL and CLC pers. obs.). In 2004, a new fisheries law (Ley de Pesca y Acuicultura No. 489) was enacted which permits the fishing of marine turtles for subsistence use only, defined as providing direct sustenance and food to the fisher and their family. The law, however, does not distinguish among sea turtle species but refers to them collectively, as a group; thus, legally allowing the subsistence use of all species. However, we do not believe this was the legislators' intent because it contradicts the Ministerio del Ambiente y los Recursos Naturales (MARENA) system of closed seasons that provides year around protection for sea turtles and their products except for the subsistence use of green turtles on the Caribbean coast [33]–[35]. In 2005, despite the 2004 fisheries law, MARENA attempted to provide protection for green turtles and their products year-around through the passage of a ministerial resolution. Each regional, autonomous government of the Caribbean coast, independently, rejected MARENA's total ban on the take of green turtles by reinstating the fishery, with the RAAS (Región Autónoma Atlántico Sur, South Atlantic Autonomous Region) reducing it by one month (August through February). Despite the closed seasons, however, the communities fish green turtles year-around, for subsistence and commercial purposes. MARENA continues to support earlier resolutions through Ministerial Resolution No. 02.01.2013 (2 January 2013), protecting all marine turtles in a year-around closed season, except for the consumption of green turtles solely for subsistence purposes by traditional green turtle fishing communities of the Caribbean coast. However, green turtles are openly butchered and the meat commercialized in the towns and communities of both Caribbean coastal regions. Thus, the contradictions and incongruencies in national and regional laws remain, and enforcement is all but non-existent.

In addition to the inconsistencies and contradictions in the law and lack of a clear mandate regarding administration of the fishery, monitoring of take levels has been irregular and inconsistent for many decades [2], [4], [16]–[18], [23], [26], [29]. The absence of continuity, resource management oversight, and implementation of regulations of this fishery has potentially far reaching affects throughout the region. Sea turtles transition through several wide ranging developmental habitats [36] and adults migrate between nesting and foraging grounds [37] that can be hundreds to thousands of kilometers apart. Foraging aggregations are known to be of mixed stock (e.g., [5], [38]–[40]) and thus the Nicaragua green turtle fishery can impact turtle populations shared by several nations. Conversely, conservation efforts enacted by other nations in the Caribbean to protect sea turtles could be diminished or annulled by overexploitation of green turtles in Nicaragua. Furthermore, a recent population assessment using survival rate estimates from turtles exposed to the Nicaragua fishery suggested that the fishery was unsustainable, raising concerns for the future outlook of green turtles in the region [41] and the artisanal fishers dependent on this resource. Other researchers have also raised concern about the potential impact of this fishery on green turtle populations in the region [42]–[45]. Local concerns for the fishery and resource resulted in the development, in collaboration with coastal communities and management authorities, of a management strategy for the green turtle fishery [46]. The goal of the strategy was to lay the ground work for development of a management plan to better regulate the fishery, and to evaluate potential alternative sources of income for green turtle fishers.

In this paper we present the first analysis of take levels and catch rate trends of the Caribbean Nicaragua green turtle fishery. Data analysis includes spatial and temporal trends for the overall fishery, by region, and at the community level for a 21-yr period (1991–2011). Trends in catch rates (this paper), and catch demographics (Lagueux et al. unpublished data) are needed to aid in assessing the status of the green turtle fishery and its potential impact on the Nicaragua green turtle foraging aggregation and green turtle rookeries throughout the greater Caribbean region that use Nicaragua's foraging grounds; and further, to provide valuable information for fishery managers to ensure sustainable resource use.

## Methods

### Ethics Statement

All necessary permits were obtained for the described study from the Nicaragua Ministerio del Ambiente y los Recursos Naturales (MARENA), and data collection complied with all relevant regulations. MARENA permits were issued to either CJL or the Wildlife Conservation Society. Data for green turtles fished from the Reserva Biológica Cayos Miskitos and the Refugio de Vida Silvestre Cayos Perlas are included in this study.

### Study Site

**Figure 2:**
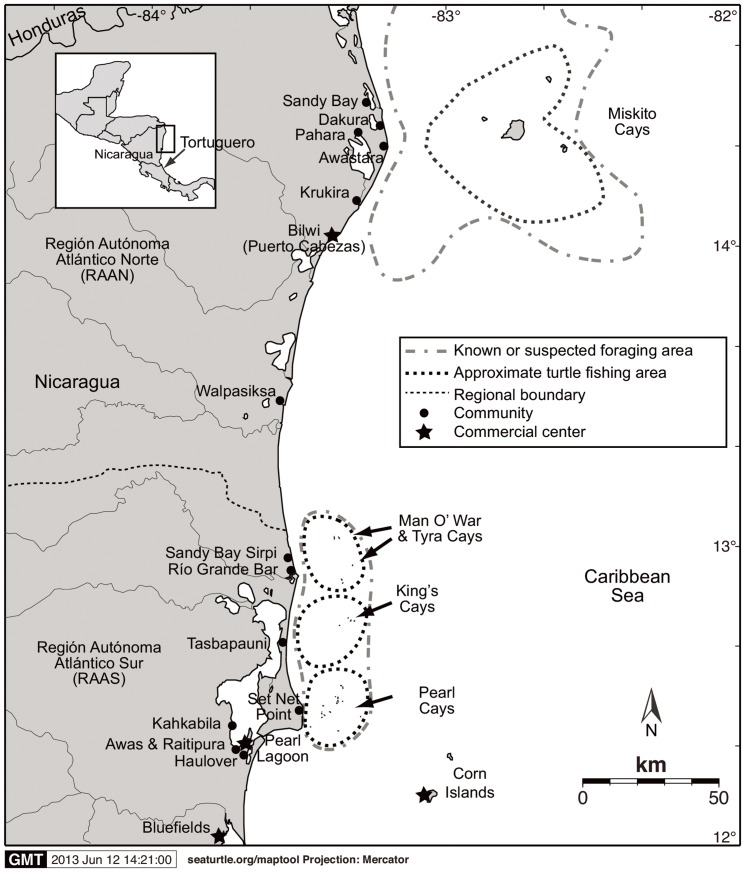
Caribbean coast of Nicaragua. Approximate green turtle, *Chelonia mydas*, foraging and fishing areas, principal green turtle fishing communities, and commercial centers; including the Tortuguero, Costa Rica, rookery. Modified from Lagueux et al. [46]. Baseline map created using SEATURTLE.ORG Maptool [109].

This study is part of an on-going, larger study of marine turtles of Caribbean Nicaragua. The fishery occurs year-around in offshore continental shelf waters of Nicaragua along the Caribbean coast in the RAAN (Región Autónoma Atlántico Norte, North Atlantic Autonomous Region) and the RAAS. Fishing locations in the RAAN are found approximately 48 km to 80 km offshore, in an area known collectively as the Miskito Cays, located approximately 70 km northeast of Puerto Cabezas, the principal coastal commercial center located in the RAAN (Fig. 2). The Miskito Cays area is comprised of several mangrove islands, and a vast area of seagrass pastures, submarine coral heads and reefs, and rock outcroppings. Fishers in the RAAN “camp” in wooden shelters constructed on pilings built over shallow water near the cays or remain on their wooden fishing dories. There is some overlap in the use of capture locations among the different communities; however, many sites are fished by a single community.

In the RAAS, principal fishing areas are found approximately 16 km to 24 km offshore from south of Prinzapolka to the Refugio de Vida Silvestre Cayos Perlas (RVSCP, Pearl Cays Wildlife Refuge) (Fig. 2). In the RAAS, fishing is conducted from temporary camps located on offshore coralline cays comprised of sandy beaches, mangroves, coconut trees and dead coral. The marine areas around many of the cays include seagrass beds; and fringing and patch coral reefs. Fishers in the RAAS use the cays and fishing grounds located east of their communities and do not travel to other community fishing grounds [18] except for some overlap among fishers from Sandy Bay Sirpi and Río Grande Bar communities and the most southern communities located in the Pearl Lagoon area which share the RVSCP fishing ground.

Green turtle fishing locations in the RAAN and RAAS are separated by approximately 130 km, where no fishing occurs. Fishers in the RAAN are indigenous Miskitu Indians, however, in the RAAS, they are Miskitu Indian, Creole, or of a mixed Miskitu Indian/Creole ethnicity. Large-mesh entanglements nets are the primary method of capture used, although a few fishers in the RAAS still strike turtles with a harpoon. Entanglement nets are set over rock or coral outcroppings known as “sleeping rocks” where green turtles return at dusk to “rest” overnight after foraging during the day. As green turtles ascend to the surface at night to breath they may become entangled in a net. Nets are checked at dawn and captured turtles are retrieved. For a detailed description of the principal technique used for catching green turtles in Nicaragua see Campbell [41].

### Green Turtle Landing Sites

In the RAAN, green turtle landings were recorded in the coastal Miskitu Indian communities of Awastara (AW), Dakura (DK), and Sandy Bay (SB); and in the commercial center of Puerto Cabezas (PC) (also known by its Miskitu name Bilwi) (Fig. 2). As a commercial center, Puerto Cabezas received landings from at least three additional communities in the RAAN, Krukira, Pahara, and Walpasiksa, however, because green turtle fishing was not a principal occupation, landings were not recorded in these communities. In the RAAS, data were recorded from the coastal communities of Awas (AS), Haulover (HH), Kahkabila (CB), Pearl Lagoon (PL), Raitipura (RP), Río Grande Bar (RG), Sandy Bay Sirpi (BS), Set Net Point (SN), and Tasbapauni (TA); and in the commercial center of Corn Island (CI) (Fig. 2). Corn Island inhabitants have almost completely discontinued turtle fishing because they earn more through lobster fishing, however, they still consume green turtles, and thus landings at Corn Island were almost entirely from RAAN or other RAAS fishers. Although green turtles are consumed in the commercial center of Bluefields, RAAS, it was not necessary to collect data there because turtles first passed through fishers communities where landings were recorded before arriving in Bluefields. A member of each community collected the fishery data except for the communities located in the Pearl Lagoon basin (AS, CB, HH, PL, and RP), where one person from the community of PL collected data for all the communities because they all fished in the RVSCP, the communities are in relative close proximity, and the commercial outlet for much of their catch was PL.

Onset of data collection, and training and supervision of community data collectors by the principal investigator (CJL) began between 1992 and 1999, depending on the site, but all continued through 2011 (except for Corn Island which ended in 2007) (Table S1). Additional green turtle landing data were also available in 1991 for PC (C. Clark, unpublished data), from April 1992 through 1993 for PC and SB (Sea Turtle Conservancy, unpublished data) and from 1991 to 1993 for BS and RG (Centro de Investigaciones y Documentación de la Costa Atlántica, CIDCA, unpublished data). Over the time period there has been relatively low turn over in data collectors; from no turn over for 10 sites (AS, CB, CI, DK, HH, PL, RP, RG, SB, and SN) to a maximum of five different data collectors for each of BS and TA.

### Types of Fishery Data Collected

Fishing trip data were recorded when a boat returned to its community, landed at a commercial center, or on the fishing grounds. The following data were recorded for each trip: 1) community, 2) landing date, 3) capture method, 4) capture location, 5) total number of green turtles captured, 6) number of nets used, and 7) number of days fished. Only turtles captured in entanglement nets were included in the catch rate analysis. For as many turtles as possible, curved plastron length (PL) was measured along the midline from the junction of the skin and intergular scute to the posterior termination of the plastron with a 150-cm flexible tape. Although PL, as an indicator of size, is not the preferred measurement for sea turtles it was the most practical measurement for community data collectors to take because animals were transported and stored on their carapace. Nevertheless, PL was converted to minimum straight carapace length (SCL) based on measurements taken on the foraging grounds in Nicaragua ([18]; CLC and CJL unpublished data). For improved accuracy separate linear equations were used to convert PL to SCL for turtles larger than 50 cm PL (SCL = 1.4109+1.2004 * PL, r^2^ = 0.86, n = 520, p<0.0001) and for those smaller than 50 cm PL (SCL = −0.7835+1.2024 * PL, r^2^ = 0.99, n = 95, p<0.0001).

### Annual Green Turtle Landings

Although the principal turtle fishing communities were monitored and catch data reported, turtle fishery data were not collected for the entire study period for all communities and not all turtles captured in the monitored communities were reported, i.e., some turtles were landed clandestinely and some trips were not recorded. None of the estimated yearly take includes green turtle landings for three additional communities in the RAAN (Krukira, Pahara, Walpasiksa) except when landed in Puerto Cabezas, by the Rama Indians located south of Bluefields, RAAS or by residents of San Juan de Nicaragua (formerly known as San Juan del Norte, located near the Costa Rica border), although take of green turtles at these sites is expected to be relatively low. Green turtles captured incidentally to other fisheries, e.g., industrial shrimp trawling, lobster or sea cucumber diving, or hook and line fisheries, were included in the estimated total take of green turtles when available, but not used in catch rate analyses. Numbers of individuals captured in these other fisheries is probably underestimated because this was not the focus of the study (Table S2). Thus, totals for annual landings are minimum take levels, although green turtle landings for some years were calculated based on the combined recorded and estimated monthly take (when data were not available). For a variety of reasons data were not collected during every month of the study period at all data collection sites, particularly early in the study. For months in which data were not available a monthly average was calculated from known months for that year and site, and the average was used to calculate an estimated monthly take for those months missing data. In 1995, however, data in the RAAN were collected for only two or three months, depending on the community, and for only 6.5 mo in PC in 2005, thus, for months in which no data were recorded landings were estimated based on the average for the same month of missing data during the year immediately prior and post the year missing data. Landings were not estimated for sites where no data for that year were available.

In the RAAN, because green turtles were often landed at more than one data collection site it was possible for animals to be recorded twice. To avoid overestimating landings, we subtracted from the total, those animals recorded at one site but sent to another site in which a data collector was employed. If turtles were sent to a site where no data collector was employed, however, then these animals were included in the total.

### Trend in Catch Rates

To assess changes in catch rates (effectively catch per unit effort, CPUE) we used green turtles caught per fishing trip as a dependent variable and the number of nets multiplied by total fishing days as an offset term in the models. We examined overall changes in green turtle catch rates over time for the fishery (the TIME corresponding to the number of days since the first fishing trip was recorded). We also assessed whether catch rates varied: seasonally (with the MONTH variable ranging from 1 to 12), regionally (with REGION corresponding to either RAAN or RAAS), and by fishing community (COM corresponding to the separate communities included in the data set). Potential interaction between TIME and either REGION or COM was also considered.

Analyses included the following three principal areas of focus:

- Overall analysis of catch rates for the principal green turtle fishing communities, comprising 10,202 records, of which 4,614 were from RAAN communities: AW (2,313), DK (900), and SB (1,401); and 5,588 were from RAAS communities: BS (2,228), RG (533), SN (483), and TA (2,344), all with good temporal coverage. Maximum time span of data analyzed covers 14 January 1991 through December 2011 with the TIME variable ranging from 0 to 7,660 days. Data collection for BS and RG began in 1991 and for SN and TA in 1995. This analysis included comparison of models with and without REGION and COM as explanatory variables.
- An analysis of catch rates for the PC commercial center for trips originating from the AW community. Only for this community was a sample size of 1,887 adequate for a separate trend analysis, with an unknown degree of overlap with data collected in the AW community. These data covered the time period 1 December 1995 to 28 December 2011 with the TIME variable ranging from 0 to 5,871 days with good temporal coverage. The purpose of this analysis was to compare the trend in catches for green turtles recorded from the AW fishers in the commercial center compared to data recorded in the AW community, hypothesizing that the catch rate trends should be similar.
- An analysis of catch rates for the Pearl Cays fishing area in the RAAS, where a wildlife refuge was established in late 2010. Currently, no management plan exists for the refuge, and thus it would be helpful to inform management plan development and to assess whether refinements to the protected area might improve its future efficacy in terms of permitted fishing activities. Communities that use the RVSCP and had a sufficiently large sample size included SN, PL, CB, and HH. The data set comprised 1,126 records from SN (483 records, beginning December 1995), PL (184 records, beginning August 1998), CB (321 records, beginning September 1998), and HH (138 records, beginning July 1999) with reasonable sample sizes and good temporal coverage. Maximum time period for these data ranged from 7 December 1995 to 31 December 2011 for which data were available from these communities, with the TIME variable ranging from 0 to 5,868 days. This analysis included comparison of models with and without COM as an explanatory variable.

For all catch rate analyses, we retained only catch data where fishing effort (number of nets used multiplied by total fishing days) and community were recorded. This is already reflected in the previously stated record counts.

Generalized Additive Mixed Models (GAMMs) were used due to their flexibility and capacity for non-linear responses (clearly evident in these data), data not collected according to a balanced design, and options for dealing with heterogeneity or temporal correlation in the counts [47]. Analyses were completed using R software [48]. The models were fitted using the gamm function from the mgcv library [49], which calls the appropriate routine in the MASS library [50]. The mixed model approach also allowed us to quantify the uncertainty associated with the smoothing parameters. The auto-correlation function allowed us to visually ascertain the degree of temporal correlation in the data and was treated using an autoregressive model of order one (AR-1) (from the nlme library for R [51]). Residuals were nested within year to speed up computation and avoid numerical problems during model fitting. Model comparisons were based on Akaike's Information Criterion (AIC). Model diagnostics (residuals vs. linear predictor, histogram of residuals, response vs. fitted values, etc.) and the statistical significance of the terms in the model (based on the approximate p-values produced by GAMM) were also considered. Cubic regression splines were used to fit the smooth function for TIME with a cyclic smooth used for the MONTH variable to ensure that the first month matched up with the last.

When investigating whether catch rates differed significantly by REGION or at the COM level, these variables were added as a factor variable. In addition, these models were contrasted to models with separate smooth functions conditioned on REGION and COM for the TIME variable to investigate the potential interaction between TIME and these other variables with either factor added as a main effect as well. When modeling the catch rates over time the results produced by using a Poisson distribution with a log-link were compared to those produced by a negative binomial distribution and log-link to ensure that potential over-dispersion in the count data were appropriately modeled. To use an appropriate value for the scale parameter theta of the negative binomial, the gam function from mgcv was run treating theta as unknown and specifying an interval of (1,7) over which to search for its value, which was rounded and applied in the corresponding GAMM model.

## Results

### Green Turtle Take Levels

**Figure 3:**
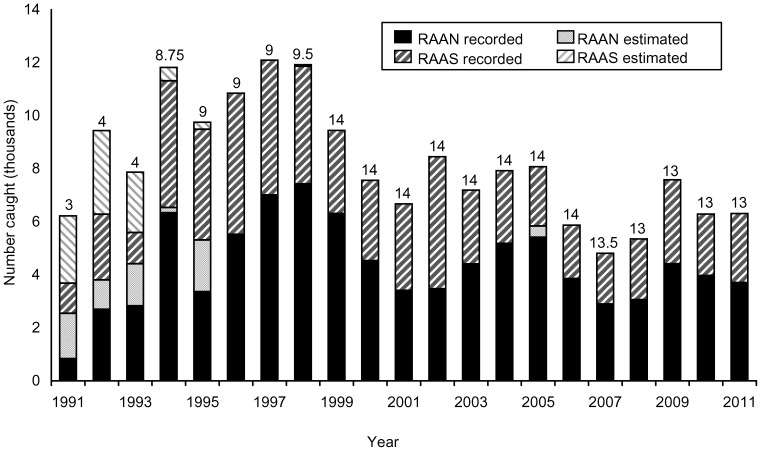
Recorded and estimated annual green turtle take by region for the Caribbean coast of Nicaragua. Numbers above bars are number of landing sites monitored each year. RAAN  =  Región Autónoma Atlántico Norte, RAAS  =  Región Autónoma Atlántico Sur. See Table S1 for information on which sites were monitored each year and Table S2 for minimum recorded and estimated annual take by community.

From 3 (1991) to 14 (1999 to 2006) sites were monitored from 1991 to 2006, and there afterward, 13 sites were monitored through 2011 (Table S1). During this 21-yr period, temporal coverage ranged from 61.9% for communities located in the Pearl Lagoon basin to 96.8% for Sandy Bay Sirpi (Table S1). Recorded take was 155,762 green turtles over 21 years, with an overall decreasing but cyclic seasonal trend since the highs of the 1990s (mean = 7,417±2,604 green turtles/yr, range = 1,967 to 12,094 green turtles (corresponding to the years 1991 and 1997, based on three and nine sites monitored, respectively)) (Fig. 3; also see Table 1). For the same 1991 to 2011 time period, total minimum take (including estimated take) was 171,556 green turtles (mean = 8,169±2,182 green turtles/yr, range = 4,812 (2007) to 12,094 (1997) green turtles. For the five year period between 1994 and 1998, when nine sites were monitored, estimated minimum mean annual take was 11,291±985 green turtles (range = 9,757 (1995) to 12,094 (1997) green turtles. For the 13 year period from 1999 to 2011, when the maximum number of sites was monitored (13 or 14 sites per year), minimum mean annual take (with less than 0.5% estimated for this period) was 7,044±1,312 green turtles (range = 4,812 (2007) to 9,448 (1999) green turtles. From 1994–2011, RAAN communities annually captured more green turtles than RAAS communities, except during 1995 and 2002. Annual take of green turtles by community and year is provided in Table S2. Although overall green turtle take decreased over time, there were two time periods where take declined more precipitously, from 1998 to 2001 and from 2005 to 2008 (Fig. 3). Average annual CPUE by community and region are presented in Table S3, and overall show declines at both community and regional levels.

**Table 1:** Recorded and estimated green turtle landings by region from the Caribbean coast of Nicaragua, 1991–2011.

1991–2001	Year	1991	1992	1993	1994^a^	1995^a^	1996^a^	1997^a^	1998	1999	2000	2001
**Recorded**	**RAAN^b^**	833^c^	2,699^d^	2,829^d^	6,338	3,364	5,522	7,009	7,425	6,315	4,532	3,412
	**RAAS** e	1,134^f^	2,482^f^	1,174^f^	4,780	4,177	5,330	5,085	4,437	3,133	3,035	3,261
	**Total**	1,967	5,181	4,003	11,118	7,541	10,852	12,094	11,862	9,448	7,567	6,673
**Estimated**	**RAAN^b^**	1,715	1,107	1,589	199	1,946	0	0	0	0	0	0
	**RAAS** e	2,541	3,152	2,278	507	270	0	0	67	0	0	0
	**Total**	4,256	4,259	3,867	706	2,216	0	0	67	0	0	0
	**Annual Total**	**6,223**	**9,440**	**7,870**	**11,824**	**9,757**	**10,852**	**12,094**	**11,929**	**9,448**	**7,567**	**6,673**

2002–2011	Year	2002	2003	2004	2005	2006	2007	2008	2009	2010	2011	Grand Total^g^
**Recorded**	**RAAN^b^**	3,471	4,409	5,184	5,414	3,853	2,897	3,057	4,415	3,973	3,703	90,654
	**RAAS** e	4,982	2,787	2,741	2,240	2,023	1,915	2,296	3,171	2,319	2,606	65,108
	**Total**	8,453	7,196	7,925	7,654	5,876	4,812	5,353	7,586	6,292	6,309	155,762
**Estimated**	**RAAN^b^**	0	0	0	423	0	0	0	0	0	0	6,979
	**RAAS** e	0	0	0	0	0	0	0	0	0	0	8,815
	**Total**	0	0	0	423	0	0	0	0	0	0	15794
	**Annual Total**	**8,453**	**7,196**	**7,925**	**8,077**	**5,876**	**4,812**	**5,353**	**7,586**	**6,292**	**6,309**	**171,556**

See Methods for a description of how estimates were calculated and Table S2 for recorded and estimated annual take by community.

a Data source from 1994 to April 1997: Lagueux [18].

bRegión Autónoma Atlántico Norte (North Atlantic Autonomous Region).

c Data source: Cecil Clark, Puerto Cabezas, Nicaragua.

d Data source for 1992 and 1993: Sea Turtle Conservancy, Gainesville, FL (formerly Caribbean Conservation Corporation).

e Región Autónoma Atlántico Sur (South Atlantic Autonomous Region).

f Data source: Centro de Investigaciones y Documentación de la Costa Atlántica (CIDCA), Bluefields, Nicaragua.

g Grand Total  =  sum of 1991–2011.

Principal life stages captured in the fishery include large juveniles and adults. Mean curved plastron lengths for the RAAN and RAAS were 72.8±6.56 cm (range = 24.5 to 99.5 cm, n = 45,130) and 69.5±9.71 cm (range = 10.1 to 99.3 cm, n = 41,442), respectively. Based on predicted SCL, mean size for green turtles captured in the RAAN and RAAS was 88.8±7.91 cm (range 28.7 to 120.9 cm, n = 45,130) and 84.7±12.0 cm (range 11.4 to 120.6 cm, n = 41,442), respectively.

### Trends in Catch Rates

Turtle catch count data were over-dispersed and thus a negative binomial distribution and log-link were used to model catch rates over time. This was confirmed by visual inspection of the frequencies of green turtles caught per fishing trip for each of the catch data sets considered (Fig. S1). Temporal correlation varied by dataset (e.g., Fig. S2) and only sometimes was it necessary to use an autoregressive model to account for this, as detailed below.

**Figure 4:**
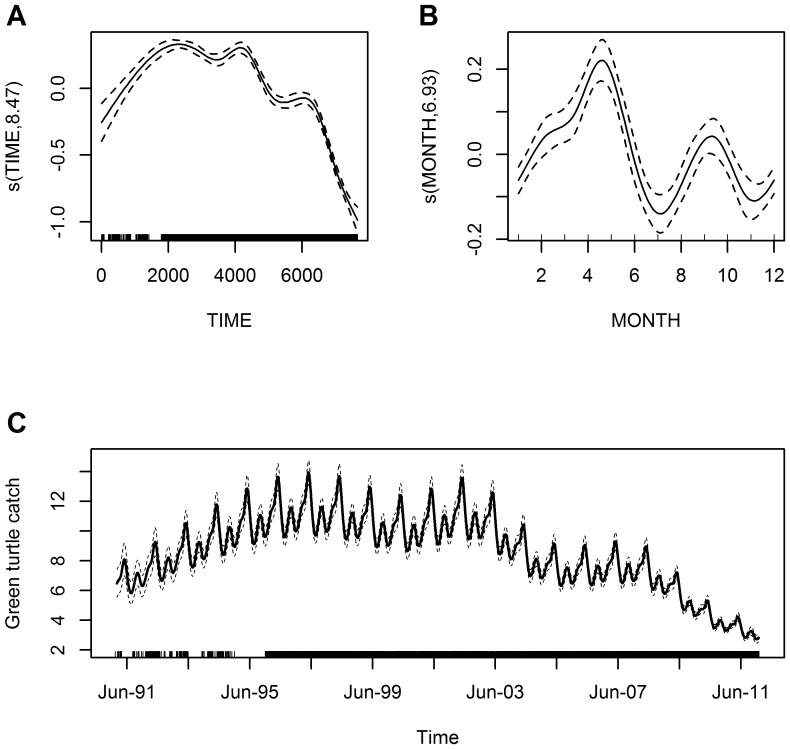
Overall trend in green turtle catch rates for the Caribbean coast of Nicaragua. Estimated conditional dependence of catch rates from 1991 to 2011 for (A) time and (B) month. Plot components are: estimates on the scale of the linear predictor (solid lines) with the y-axis scale for each variable selected to optimally display the results, confidence intervals (dashed lines), and explanatory variable values of observations shown as a rug plot along each x-axis. Also shown on the scale of the response is (C) trend in catches (average turtles/day) over time from 1991 to 2011, assuming average fishing effort for this period in terms of nets used and trip length. The seven principal fishing communities included in the analysis are: Awastara, Dakura, and Sandy Bay in the RAAN; Río Grande Bar, Sandy Bay Sirpi, Set Net Point, and Tasbapauni in the RAAS.

Overall trend in catch rates based on the data from the seven principal green turtle fishing communities fluctuated seasonally and peaked in mid-1997 and again in 2002 with a declining trend in catch rates from that point in time onwards. From mid-2008 there was a dramatic decline that continued to the end of the study period (Fig. 4A and C). Over the study period, seasonal variation (MONTH) in catch rates peaked in April-May and to a lesser degree in September-October, and dropped-off significantly in July and November (Fig. 4B), with temporal correlation not posing a severe problem (Fig. S2A).

**Figure 5:**
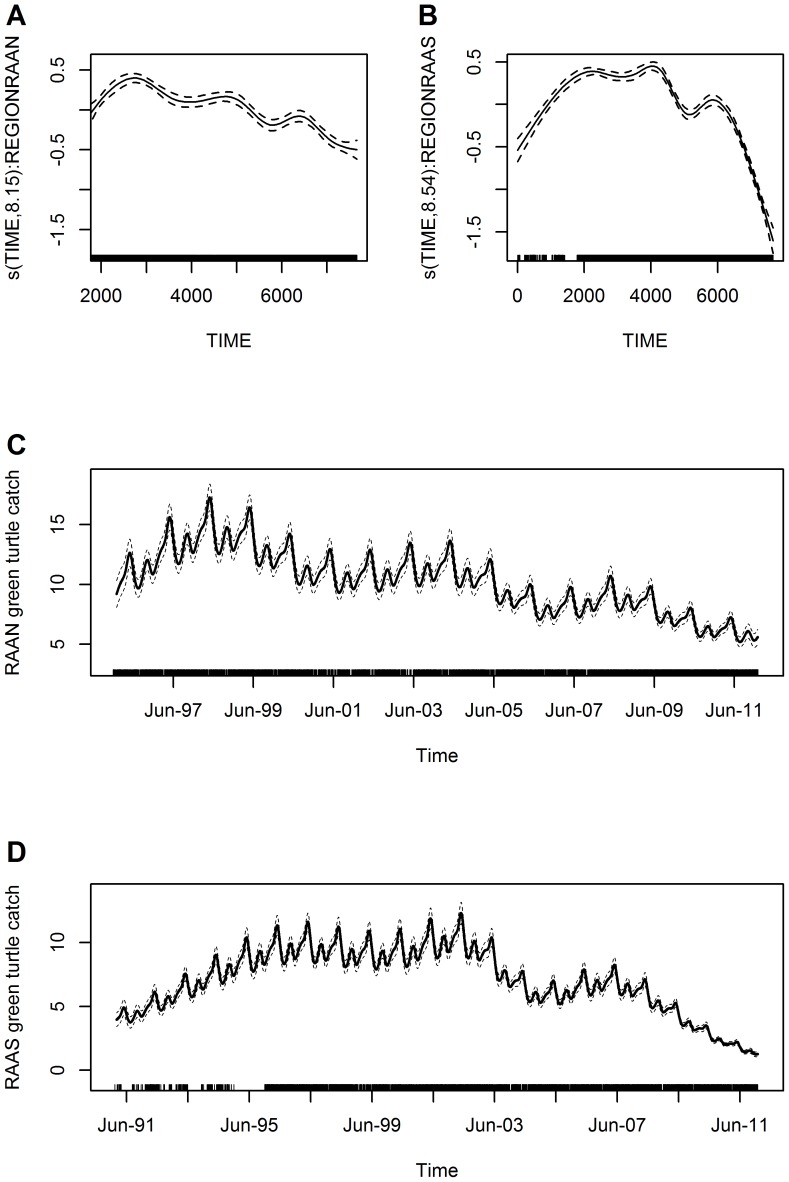
Regional level trends in green turtle catch rates for the Caribbean coast of Nicaragua. Estimated conditional dependence of catch rates on time by (A) RAAN  =  Región Autónoma Atlántico Norte and (B) RAAS  =  Región Autónoma Atlántico Sur. Seasonality is also included in the regional models, but the corresponding plot is not shown, as it is indistinguishable from that shown in Fig. 4B. Plot components are the same as for Fig. 4A and 4B. Note that the y-axis scale is the same for both Fig. 5A and 5B. The x-axis scale corresponds to the time periods when sampling took place (December 1995 to 2011 in the RAAN and 1991 to 2011 in the RAAS). Also shown on the scale of the response are trend in catches (average turtles/day) over time (C) in the RAAN and (D) in the RAAS, assuming average fishing effort by region in terms of nets used and trip length for those time periods. The analysis is based on data from the same seven principal fishing communities included in Fig. 4.

For the years 1991 to 2011, estimated catches (per average effort fishing trip in terms of nets used and trip length) for the overall trend model declined from 6.5 to 2.8 green turtles, which corresponds to approximately a 56% decline over the 21-year period (Fig. 4C). The regional trend model showed the same seasonality and again temporal correlation did not pose a severe problem (Table 2; Fig. 5). The RAAN model estimated an approximately 39% decline, from 9.2 to 5.6 green turtles caught per average fishing trip, for the shorter 1995 to 2011 time period (Fig. 5C). The RAAS model estimated an approximately 85% decline, from 8.8 to 1.3 green turtles caught per average effort fishing trip, for the same time period (Fig. 5D). When including the entire 1991 to 2011 sampling period for the RAAS the decrease was approximately 68% with an estimated average of four green turtles caught at the beginning of 1991, however, results from 1991 to November 1995 were based on only two communities, BS and RG (Fig. 5D). Nevertheless, this indicates that catch rates have dropped more dramatically in the RAAS green turtle fishing areas.

**Table 2:** Generalized Additive Mixed Model (GAMM) analysis results for green turtle catch data from the Caribbean coast of Nicaragua.

	COM		AW COMCTR		PCFA		Explanatory Variables					
Analysis Type	AIC	Δ AIC	AIC	Δ AIC	AIC	Δ AIC	TM	MN	RG	CM	TIME By	corAR1^a^
Overall	24,286	5,314	3,308	6	3,330	626	***;***;***					
	24,359	5,387	**3,302**	0	3,350	646	***;***;***					X
	***23,894***	4,922	3,310	8	***3,275***	571	***;***;***	***;NS;***				
	23,980	5,008	3,304	2	3,299	595	***;***;***	***;NS;***				X
Regional	22,426	3,454	-	-	-	-	***;-;-		***;-;-		RG	
	***22,147***	3,175	-	-	-	-	***;-;-	***;-;-	***;-;-		RG	
	22,160	3,188	-	-	-	-	***;-;-	***;-;-	***;-;-		RG	X
Community	19,205	233	-	-	2,757	53	***;-;**			***;-;***	CM	
	19,090	118	-	-	**2,704**	0	***;-;***	***;-;***		***;-;***	CM	
	**18,972**	0	-	-	2,713	9	***;-;**	***;-;***		***;-;***	CM	X

For each model its AIC value and the difference in AIC values between the top ranked model (value in bold) and other models (**Δ**AIC) is shown. Models are sorted by analysis type: overall, regional (when applicable), and community level (with the “best” model in terms of smallest AIC value in ***bold italics*** by analysis level and in **bold underline** overall).

Results are shown for the three data sets analyzed: COM  =  principal fishing community data, AW COMCTR  =  commercial center data for the Awastara community, PCFA  =  Pearl Cays fishing area data. Explanatory variable acronyms are: TM  =  TIME, MN  =  MONTH, RG  =  REGION, CM  =  single or aggregated community, TIME by  =  interaction between TIME and either REGION or community.

For terms included in a model, p-values were indicated as follows: *** = <0.001 or ** = <0.01 and values >0.1 are designated as non-significant (NS), with results for the three data sets considered separated by a semi-colon.

a “X” indicates when temporal autocorrelation was treated using an autoregressive model of order one.

**Figure 6:**
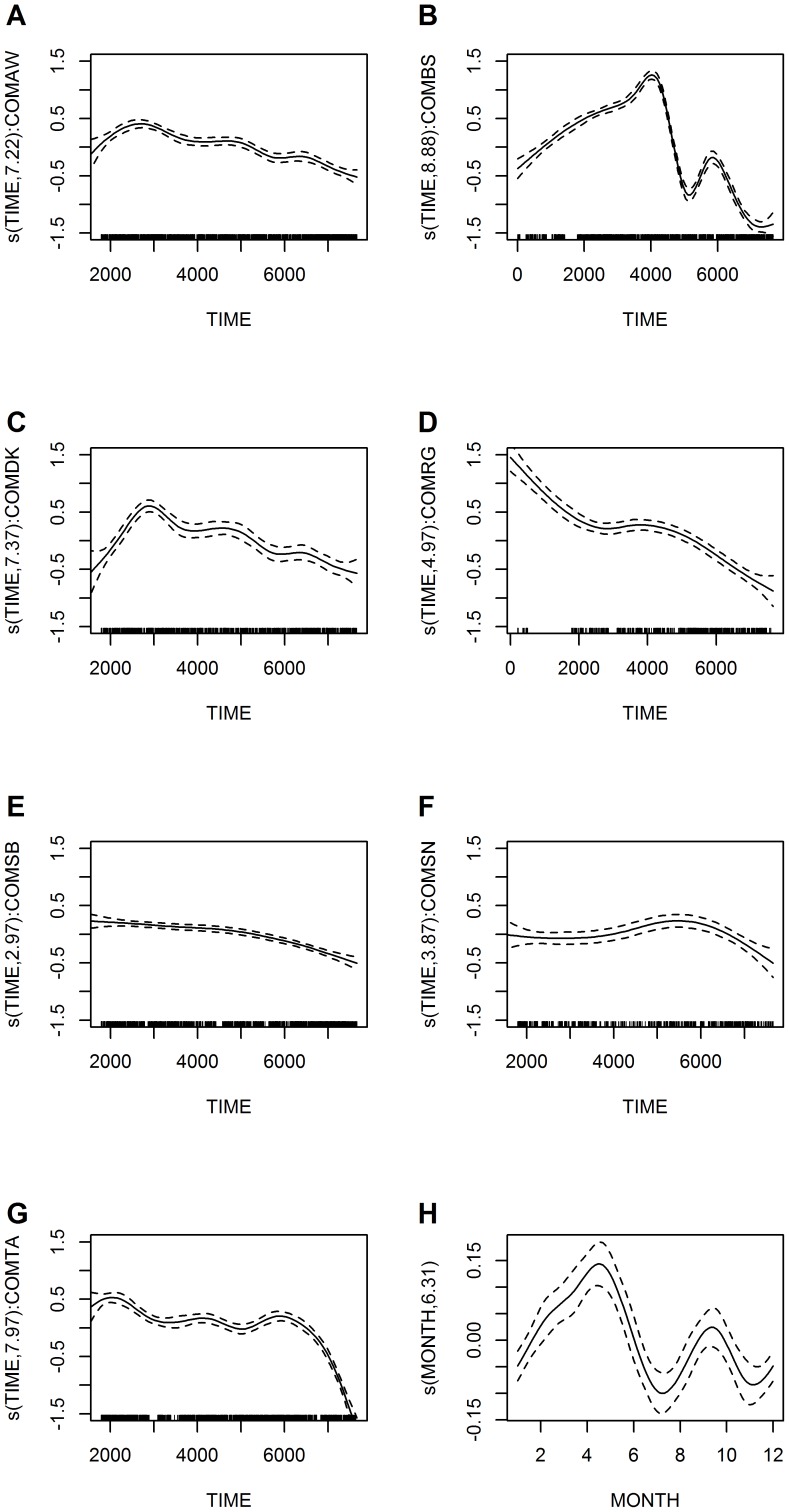
Community specific trends in green turtle catch rates for the Caribbean coast of Nicaragua. Estimated conditional dependence of catch rates on time by communities in the RAAN (A) AW (Awastara), (C) DK (Dakura), and (E) SB (Sandy Bay), and in the RAAS (B) BS (Sandy Bay Sirpi), (D) RG (Río Grande Bar), (F) SN (Set Net Point) and (G) TA (Tasbapauni). Seasonality is also included in the community level model, and although the seasonal pattern in catch rates is the same as for the overall and regional models, the variance explained by seasonality is much less than before (as shown in Fig. 6H) when considering catch rates at the community level. Plot components are the same as in Fig. 4A and 4B. Note that the same y-axis scale is used for each variable (with the exception of month). The x-axis scale corresponds to the time periods when sampling took place (December 1995 to 2011 in all communities, except BS and RG in the RAAS where sampling started in 1991).

For community specific trends, the best model in terms of AIC value was the autoregressive model that dealt explicitly with temporal correlation (Table 2; Fig. 6), and showed the same pattern in seasonality (Fig. 6H), although this variable was less influential than in the overall or regional models. For the purpose of comparison, we focused on the December 1995 to 2011 time period during which data were available for all communities. Catch rate trends showed an overall decrease (AW 45%, DK 21%, SB 52%, BS 83%, RG 73%, SN 38%, and TA 90%), assuming community specific average fishing effort in terms of nets used and trip length (Fig. 6A–G). This corresponds to the following number of green turtles caught per average community fishing trip at the beginning and end of the period in the RAAN: 10.9 and 6 (AW), 4 and 3.1 (DK), 13.5 and 6.5 (SB), and in the RAAS: 7.4 and 1.3 (BS), 19 and 5.2 (RG), 5.1 and 3.2 (SN), and 9.3 and 1 (TA) (Fig. S3). However, AW, DK, BS, and TA showed initial increases in catch rates with BS having the longest extended increase in catch rates before it began a drastic decline in December 2001.

**Figure 7:**
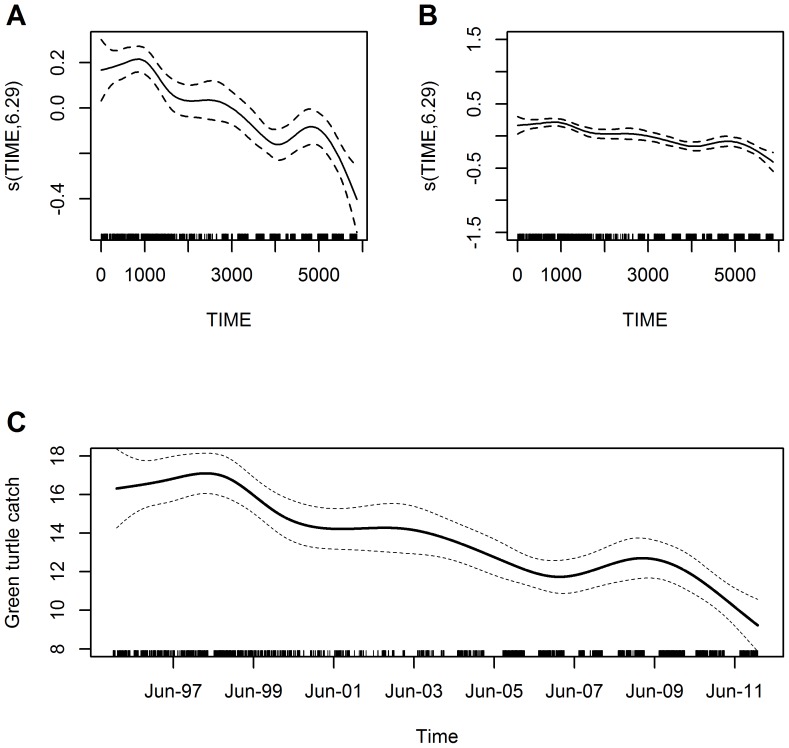
Trends in green turtle catch rates for Awastara (AW) landings in Puerto Cabezas (PC). Estimated conditional dependence of catch rates on time (A) with the y-axis selected to optimally display the results and (B) with the y-axis scale the same as for Fig. 6A to facilitate comparison. Plot components are the same as in Fig. 4A and 4B. Also shown on the scale of the response is (C) trend in catch rates (average turtles/day) over time from 1996 to 2011, assuming average fishing effort for AW in terms of nets used and trip length for the period.

Model results for catch data recorded in the PC commercial center that originated from AW fishers show a similar pattern as results for data recorded in the AW community. Results were improved in terms of AIC value by not including seasonality and using an autoregressive model that dealt explicitly with temporal correlation (Table 2; Figs. 7 and S2B). For these data, the trend model for the years 1996 to 2011 estimated that catch per average effort fishing trip in terms of nets used and trip length declined from 16.3 to 9.2 green turtles, which corresponds to approximately a 44% decline over a 16 year period (Fig. 7C), compared to a 45% decline estimated by the model for data recorded in the community (Fig. S3A).

**Figure 8:**
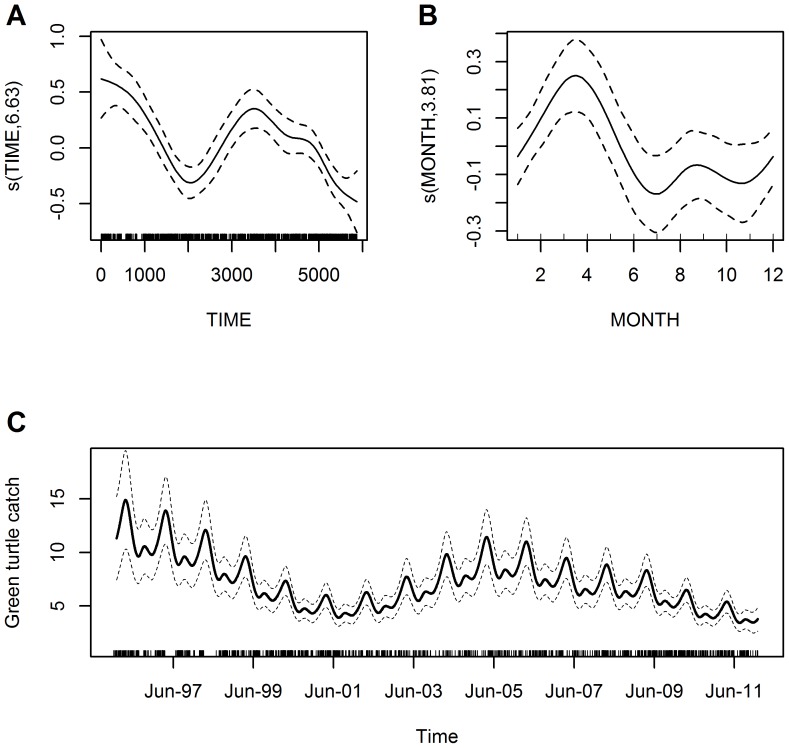
Overall trends in green turtle catch rates for communities fishing in Refugio de Vida Silvestre Cayos Perlas. Estimated conditional dependence of catch rates on (A) time and (B) month. Plot components are the same as in Fig. 4A and 4B. Also shown on the scale of the response is (C) trend in catch rates (average turtles/day) over time from 1996 to 2011, assuming average fishing effort across the four communities in terms of nets used and trip length. The four communities included in the analysis were: Haulover, Kahkabila, Pearl Lagoon, and Set Net Point.

Overall trend in catch rate data for the communities that used the RVSCP fishing area showed a decline similar to previous results. In contrast, both seasonal variation peaks occurred a month earlier than reported above (March-April and to a lesser degree again in August-September), although they dropped off significantly in July and November (same for results reported above), and temporal correlation did not pose a severe problem (Table 2; Figs. 8 and S2C). Overall trend model for years 1996 to 2011 estimated that catch per average effort fishing trip in terms of nets used and trip length declined from 11.3 to 3.8 green turtles, an approximately 67% decline over a 16 year period (Fig. 8C).

The best community specific trend models included seasonality (Fig. S4). Although important, the seasonality pattern was less pronounced for the RVSCP fishing communities (Fig. S4E) compared to the overall trend (Fig. 4B), and again temporal correlation did not pose a severe problem (Table 2; Fig. S4). For the purpose of comparison, we focused on the 1999 to 2011 time period during which data were available in all the communities using the RVSCP fishing area. For CB, PL and SN, assuming community specific average fishing effort in terms of nets used and trip length, the model estimated declines in catch rates of approximately 13%, 50%, and 26%, respectively. Although HH showed an overall increase of 69% due to very low initial catch rates, the recent trend shows a decline. Changes in green turtles caught per average community fishing trip were 7.1 to 6.2 (CB), 7 to 3.5 (PL), 5.5 to 4.1 (SN, Fig. S4F), and 4.4 to 7.4 with a peak of 14.9 in mid-2006 (HH). Due to small sample sizes, especially for CB, HH, and PL, these community specific results should be interpreted with caution.

All models considered indicated a statistically significant change in catch rates over time, with the exception of the last model described where the community specific trends for CB, HH, and PL do not provide much support of a temporal trend in catch rates (likely due to sample size issues for these three Pearl Cays fishing area communities).

## Discussion

### Take Levels

The Caribbean Nicaragua artisanal, commercial green turtle fishery is currently the second largest legal turtle fishery in the world (see Humber et al. [52]), with a mean estimated minimum take of 8,169±2,182 green turtles per year during the past 21 years (this study). Annual take of green turtles from the mid to late-1990s (this study) was similar to or exceeded annual exploitation levels reported from the late 1960s to early 1970s [16], when evidence of declines in the foraging aggregation in Nicaragua and the rookery at Tortuguero were attributed to overexploitation [16], [24], [28], [30], [53]. Indications that the population was in decline at that time were: 1) a decrease in catch rates from one turtle per two person-days in 1971 to one turtle per six person-days in 1975 [16], [24], [30], 2) a decrease in the capture of larger turtles [16], [28], and 3) a severe decline in the nesting density of females at the Tortuguero rookery in the late 1960s-early 1970s [53].

Current take levels are much higher than several decades ago as evidenced by take recorded for two communities in the RAAS where historical landings data are available. In Tasbapauni, during the second half of the 1990s, there was a two- to three-fold increase in the take of green turtles compared to just prior to the operation of green turtle processing plants in Nicaragua. For only two years between 1994 and 2011 were take levels in Tasbapauni (Table S2) less than the take reported for a 12-month period beginning in 1968 [16], [28]. For Sandy Bay Sirpi, take levels were higher for six of the years between 1992 and 2011 (Table S2) than reported for a 12-month period beginning in 1972 [24], a period during processing plant operations.

Furthermore, annual recorded take of green turtles from the Miskito Cays area (RAAN) were higher for all years between 1994 and 2011 (post Sandinista/Contra war) than for mean annual take from 1985 to 1990 (a period including the Sandinista/Contra war and immediate post-war), when an estimated average 2,783±682 green turtles/yr (range = 1,619 to 3,383 turtles/yr, n = 6 yrs) were taken [17]. During the 1990s, minimum average annual green turtle take for the Miskito Cays area was 4,704±2,318 (range = 833 to 7,425 turtles/yr, n = 9 yrs), and during the 2000s, average annual take was 4,064±865 green turtles (range = 2,897 to 5,414 turtles/yr, n = 10 yrs).

### Protection (laws and events)

Recent increased take levels observed in this study were due in large part to increased demand for inexpensive protein from a growing coastal population and need for income generating activities. However, the increased take was likely only possible due to several major events that occurred in Costa Rica and Nicaragua during the early 1960s, and late 1970s and 1980s, which resulted in a decrease in take levels in both countries during those periods. The earlier decrease in take allowed for some recovery of green turtle rookeries impacted by the fishery (including the Tortuguero rookery) and thus, foraging aggregations increased for a few decades, which enabled high take levels during the 1990s. In 1963, Costa Rica banned the collection of eggs and killing of nesting females at the rookery (Executive Decree No. 9) and in 1975, established Tortuguero National Park to protect breeding animals in nearshore waters, and nesting females and their eggs (Law No. 5680). By 1978, the two turtle packing plants in Costa Rica had closed [21]. This was significant to the Nicaragua foraging aggregation because 1) based on tag recoveries and satellite tracking of nesting females from Tortuguero the majority use the Nicaragua foraging grounds [21], [44] and 2) based on mitochondrial DNA analysis the majority of adult animals foraging in Nicaragua are from the Tortuguero rookery [54]. It is important to note that the mtDNA analysis conducted thus far used the shorter sequences that are inadequate to distinguish among many of the rookeries in the region, thus, a study using longer mtDNA sequences on the foraging aggregation in Nicaragua should provide a much higher resolution of the rookeries impacted by this fishery.

The lower take of green turtles in Nicaragua during the 1980s was undoubtedly, at least partially, the result of civil unrest and military conflict created by the Sandinista/Contra war. Many people from coastal and interior Caribbean communities were displaced during the war, and government and private infrastructure was destroyed or severely crippled, which likely reduced the exploitation of terrestrial and aquatic resources [9], [55], [56]. According to local informants, many coastal communities were abandoned during the war; in the RAAN, only fishing trips that originated from Puerto Cabezas were permitted by the Sandinista military; and during times of heightened military activity offshore fishing was either too dangerous or not allowed along the entire Nicaragua Caribbean coast [9], [57].

The reduced take levels of marine turtles from the high levels of the late 1960s and early 1970s, provided approximately 15 or more years for some segments of the population (large juveniles and adults) to increase. Long-term monitoring of trends in nesting levels at green turtle rookeries have documented increases, once the principal threat(s) was reduced or eliminated (see Seminoff [42] for an overview but also e.g., Balazs and Chaloupka [58], Broderick et al. [59], Chaloupka et al. [60]). With the end of the Sandinista/Contra War, people returned to their communities or moved to coastal towns instead of their communities (CJL pers. obs.). It would have taken fishers some time to reestablish themselves in their communities, form a fishing crew, procure supplies and materials necessary for turtle fishing, restore their fishing skills, and/or train new crew members. Many young adult males would not have had the opportunity to learn fishing skills in their teens or as young adults due to the displacement of their families and communities from traditional fishing areas. Together with the recovery of green turtles on the foraging ground and females at the nesting beach these impediments provided an even longer reprieve and would explain, at least in part, why take levels in Nicaragua could be as high as they were from the mid-1990s to early 2000s.

### Changes in Catch Rates

Declines in take levels alone (Table 1; Fig. 3) do not necessarily indicate a decrease in turtles available to the fishery, and hence the need to assess catch rates, which incorporate fishing effort. The GAMM models, however, also indicate statistically significant declines in green turtle catch rates across the entire fishery. The peak in catch rate in 2002 was followed by an overall declining trend, even though there were seasonal fluctuations, that accelerated dramatically from 2008 and continued to 2011 (Fig. 4A and C). Thus, the overall decline in catch rates on the foraging ground might suggest the foraging aggregation is in decline, however, the green turtle rookery assumed to be most impacted by the fishery, at Tortuguero, Costa Rica, has not shown signs of decline but rather an increasing trend in nesting emergences [61], [62]. It is worth noting that although Tortuguero rookery nest counts continue an upward trend the rate of increase since 2001 has slowed (see Troëng and Rankin [62] Fig. 2B, [63]), rather than a more rapid increase that might be expected for a recovering turtle population (e.g., green turtles - Florida [60], Kemp's ridley - Mexico [64], hawksbills - Barbados [65]). Nevertheless, Bjorndal et al. [61] and Troëng and Rankin [62] call for caution when interpreting their results because i) they are based on nesting emergences, which do not necessarily translate into an increase in the number of nesting females because clutch frequency may vary; ii) their trend analysis does not provide information on hatching success, hatchling survival, or abundance and survival rates of the numerous juvenile cohorts and males that comprise other segments of the population; and iii) environmental factors very likely influence remigration intervals [66], [67] which affect estimates of female population size. Additionally, whether or not emergences actually resulted in egg deposition was not verified during the nesting beach surveys nor was the accuracy of the surveyors assessed. Nevertheless, the increasing trend in nesting activity at Tortuguero suggests an increase in females, which could be a reflection of decreased take levels in Nicaragua in the late 1970s and 1980s.

We consider several factors that might influence a decrease in take levels and catch rates in Nicaragua other than depleted stocks from overfishing: i) turtles may have changed their use of the seagrass habitat in fishing areas on Nicaragua's continental shelf such that turtles are less susceptible to capture, possibly due to reduced abundance and/or quality of forage, ii) fishers are less proficient at capturing turtles due to an increase in less experienced men in the fishery, and/or iii) due to the fishing technique, fishing has disproportionately selected turtles that use “sleeping rocks” (see Lagueux [18]) rather than those green turtles that may not use them, thus not necessarily depleting the stock but affecting the catchability of turtles at “sleeping rocks” in the future. Green turtles improve the intake quality of forage by feeding on the base of *Thalassia testudinum* leaves, and by maintaining cropped plots of turtle grass which results in higher nitrogen and lower lignin content of the forage and increased turtle growth rates [68], [69]. At this time there is no evidence to suggest that turtles have shifted or emigrated from the Nicaragua foraging grounds or that the forage is of lower quality, although with potentially fewer green turtles on the foraging ground there are less turtles to maintain cropped seagrass plots and thus, quality of the forage should be investigated. Similarly, it seems highly unlikely that fishers are less proficient in capturing turtles since sufficient time has passed for them to have regained or learned turtle fishing skills between the end of the 1980s civil war and the decline in catch rates observed beginning in 2002. In contrast, it is likely that not all green turtles use “sleeping rocks”, and if using these areas is an innate trait rather than a learned behavior, it is possible that the fishery is inadvertently reducing the number of turtles using “sleeping rocks” by reducing their survival probability.

Possible explanations for the apparent discrepancy between the increasing trend in nesting emergences at Tortuguero and decreasing catch rate trends in Nicaragua are:

- increased reproductive output (e.g., shorter remigration intervals, increased clutch frequency, and/or shorter age to maturity) resulting from reduced competition for food resources due to declines in green turtle populations throughout the Caribbean [6], [70];
- insufficient time has passed to observe the impact of the fishery on the rookery due to a time lag;
- changes in other segments of the population have not been detected since only nesting activity is monitored;
- the expansive northern Nicaragua foraging ground, which is not entirely subjected to turtle fishing [43], may provide a refuge for a sufficient portion of the Torttguero rookery;
- a larger than expected contribution of non-Tortuguero rookeries occurs in Nicaragua turtle fishing areas, which may be revealed by higher resolution genetic stock analysis; and/or
- there is a more complex scenario of population ecology and fluctuating human perturbations, that is not yet fully understood.

### Regional Impacts

Dow Piniak and Eckert [71] reported 52% (308 sites) of all known green turtle rookeries throughout the wider Caribbean supported <25 crawls per year and for another 24% (142 sites) of the sites there was insufficient data to estimate annual nesting levels. The emphasis on recovering critically endangered hawksbill populations throughout the Caribbean [72]–[75], although warranted, combined with funding shortages have often detracted from maintaining at least a minimal level of effort on monitoring green turtle rookeries and foraging aggregations. Hawksbill and green turtle nesting activity often overlap spatially and temporally, and they often forage in areas that are in close proximity as well. Thus, with a minimal amount of additional funds and/or effort many remnant green turtle populations could be better monitored in the Caribbean so that changes in population levels may be detected.

Despite sparse monitoring efforts of green turtle rookeries, evidence from flipper tag returns, satellite tracking, and/or genetic analyses provides evidence that the Nicaragua green turtle fishery may be impacting other rookeries and foraging aggregations throughout the wider Caribbean, e.g., Bahamas, Bermuda, Brazil, Cayman Islands, Colombia, Costa Rica, Cuba, Mexico, Panama, Puerto Rico (Culebra), Surinam, United States (Florida), and Venezuela (Aves Island) ([44], [54], [76]–[85]; and CJL pers. obs.). Thus, unmanaged, and potentially unsustainable, turtle fisheries can have far reaching and unwanted affects.

Despite decades of conservation effort, take of green turtles and their eggs, legal or otherwise, continues throughout the wider Caribbean (for an overview see Bräutigam and Eckert [34], King [86], Fleming [87], and Humber et al. [52] but also, for example, British Virgin Islands [88], Cayman Islands [89], Grenada [90], Montserrat [91], Turks and Caicos Islands [91]–[93], and Colombia [94], [95]). The extent of the impact from these and other threats (e.g., bycatch, disease, and/or habitat alteration) on green turtle populations is not well known, because for the most part monitoring is lacking and/or the genetic composition of rookeries and mixed-stock analyses of foraging aggregations are incomplete. Thus, implementation of a management plan for sustainable take, combined with long-term monitoring and periodic assessment of the Nicaragua fishery is highly valuable to aid in developing a more sustainable fishery.

### Survival and Life History

Survival rate estimates from recent studies generally show that rates are considerably lower where human induced mortality is apparent (Table 3). For example, survival rate estimates for green turtles exposed to the fishery in Nicaragua were relatively low [43]. Lower survival rates, however, can also be primarily a result of emigration when turtles shift developmental habitats, as was likely the case for subadults studied in Culebra, Puerto Rico [85]. Nevertheless, the relatively low survival rates for green turtles on the Nicaragua foraging ground and at the principal rookery are most likely due to an unsustainable take. Nesting females from the Tortuguero rookery primarily use the RAAN foraging ground in Nicaragua (see Troëng et al. [44]) which is much larger than the RAAS foraging ground. Given the higher survival of adult females from the Tortuguero rookery, compared to survival rate of the mixed large juvenile/adult group on the RAAS foraging ground, Campbell and Lagueux [43] suggested this may be due to the size difference between the RAAN and RAAS foraging areas resulting in a wider dispersal of females in the RAAN, thus reducing their exposure to the turtle fishery. Regional catch rate results in this study indicated that RAAN rates are now more than double those of the RAAS, although both regions have declined over time, which also suggests possible differences in fishery impact on the foraging grounds, e.g., the RAAS foraging ground may be more over fished due to its smaller size and greater accessibility. Furthermore, several studies have found size classes of green turtles segregate on the foraging ground, with larger animals using deeper water habitats [96]–[98], which could also reduce the exposure of females and other turtles in the RAAN to the fishery. Regardless of the influences on higher survival rates of Tortuguero adult females, Campbell [41] determined that multiple iterations of even the most conservative population model, using a range of survival rate estimates, indicated a declining green turtle population and concluded that take levels by the Nicaragua fishery at that time were likely unsustainable.

**Table 3:** Survival rate estimates for wild populations of green turtles.

Country	Location	Size Range or Size Class (cm)^a^	Human Induced Mortality	Survival Estimate^b^	Confidence Interval (95%)	Reference
**Bahamas**	Conception Creek	20–64	Yes	0.68 (Φ)	0.63–0.73	[78]
	Union Creek	25–84	No	0.89 (S)	0.72–0.96	[78]
**Nicaragua**	Región Autónoma Atlántico Sur (RAAS)	65.9–102.0	Yes	0.66 (S)	0.51–0.79	Campbell unpubl. data updated from [43]
**Puerto Rico**	Culebra	24–65	No	0.83 (Φ)	0.79–0.87	[85]
	Culebra	65–90	No^c^	0.53^c^ (Φ)	0.39–0.67	[85]
**Costa Rica**	Tortuguero	Adult females	Yes	0.82 (S)	0.73–0.89	[43]
	Tortuguero	Adult females	Yes	0.85 (Φ)	0.83–0.87	[45]
**Australia**	Southern Great Barrier reef	40–65 CCL	No	0.88 (Φ)	0.84–0.93	[108]
	Southern Great Barrier reef	65–90 CCL	No	0.85 (Φ)	0.79–0.91	[108]
	Southern Great Barrier reef	Adult	No	0.95 (Φ)	0.92–0.98	[108]
**Mexico**	Bahía de los Ángeles	46–77.2	Yes	0.58 (S)	0.36–0.78	[96]
	Bahía de los Ángeles	Adults	Yes	0.98^d^ (S)	0.84–0.99	[96]
	Bahía Magdalena	approx 40–90	Yes	0.85 (Φ)	0.83–0.88	[97]

a Unless otherwise stated, all size data is straight carapace length measured from the midpoint of the nuchal notch to the most posterior tip of the longest posterior marginal scute. CCL  =  curved carapace length measured from the midpoint of the nuchal notch along the curve of the midline to the posterior end of the carapace.

b S = true survival; Φ = apparent survival.

c Patrício et al. [85] acknowledged estimate is likely low due to permanent emigration, and human induced mortality was considered unlikely but cannot be ruled out entirely.

d Seminoff et al. [96] cautioned about reliability of survival probability estimate due to small sample size.

Life history traits that have coevolved with longevity have resulted in populations, such as green turtles, that are severely limited in their ability to respond to increased mortality of juveniles or adults [99]. Musick [100] stated that species with these traits are vulnerable to excessive mortality, rapid population collapse, and even if the mortality is reduced are often slow to recover. Crouse et al. [101] and Crowder et al. [102] have shown population growth rate is most sensitive to changes in annual survival of large juveniles and this life-stage may be particularly critical to population maintenance and recovery. In addition, Crouse [103] and Bjorndal et al. [104] cautioned against the use of trends of later life stages, e.g., nesting females, to indicate the effect of human induced mortality on the status of younger life stages, whose affect might not be manifested for decades.

### Limitations on Turtle Fishing

Although by Nicaragua law, green turtles can only be used for subsistence purposes and it is prohibited to commercialize the meat, neither the national law nor regional autonomous government resolutions to manage the fishery are effective. Cultural taboos and/or restrictions by indigenous or ethnic coastal societies to protect against overexploitation that may have influenced fishing levels in the past, no longer exist. Under current economic and social conditions, there is little that reduces the take of green turtles, e.g., inclement weather, sometimes holidays, or an influx of cash from the sale of narcotics found in offshore waters or washed-up on the shoreline. In the past two decades, five hurricanes have made landfall on the Caribbean coast of Nicaragua, ranging in intensity from Hurricanes Cesar (1996) and Ida (2009) as Category 1 to Hurricane Felix (2007) as a Category 5 [105]. Not only hurricanes but localized tropical storms may limit the take of green turtles by the occurrence of unsuitable winds and/or currents for fishing green turtles. In the case of Hurricane Felix, because it made a direct hit on the Miskito Cays area (in the RAAN) and green turtle fishing communities, many people lost their lives, and boats and fishing equipment were destroyed or lost. As a result of this tragic event many people were afraid to return to the Miskito Cays area to fish for a period of time. Although certainly devastating to human life and property, in general, adverse weather events are relatively uncommon and their effects are short-lived, such that the overall take of turtles is little or unaffected.

The other event that can impact the take of green turtles, although localized, is the byproduct of drug trafficking between Colombia and the United States along the Central American coastline. As drugs are transported by sea northward some are reportedly discarded as payment for passage through the area or when drug interdiction authorities intervene, thus providing a quick source of cash to fishers or family members when they find cocaine floating at sea or washed-up on shore. Buyers for the discarded drugs appear in coastal towns and communities. Money from the sale of drugs is often shared among family members, community leaders, and churches. In general, people do not consider the drugs or the money that it generates as evil but as a blessing, although some have observed the devastating affects of increased drug use in their communities and despise it. With fast cash, available from the sale of drugs, men stay in their communities and do not need to go to sea to fish, providing a reprieve, albeit short-lived, for the green turtles, and other natural resources.

### Seasonality

There is evidence for seasonality in catch rates (Fig. 4B) even though fishing occurs in all months. In contrast, Nietschmann [16] reported that in one community, prior to the opening of the turtle processing plants, turtle fishers seasonally divided their time among hunting, turtling, and tending to agricultural plots, other household or community demands, and availability of turtles on the foraging ground. He reported a decrease in monthly take levels between April and July and again between September and November [16], although similar in timing, our data indicates a shorter period of reduced take occurring in July and November. Nietschmann [16] attributed the declines in take to the temporary emigration of breeding adult turtles off the foraging ground in the spring months and an increase in rainfall during both of the periods. In contrast, when the turtle processing plants opened and the demand for green turtles increased, turtle fishers extended their turtling activities year around [16], suggesting there may have been other influences (e.g., livelihood patterns) on the seasonality of turtle fishing prior to the establishment of the processing plants, and the opportunity to earn income from selling turtles took precedence over those earlier influences. Further evidence that the migration of turtles to nesting sites does not deter fishing is that migrating turtles are absent from the foraging grounds for a longer period than the length of the seasonal catch rate decrease and thus, seasonality in the fishery may be more related to local weather patterns that reduce the effectiveness of net fishing (e.g., direction and/or strength of current or wind, and quantity of effluent from watersheds, CLC pers. obs.).

### Expanding Markets

Although no longer legally exported, current local and national demands for green turtle meat within Nicaragua have grown to equal or exceed export demands for green turtle products that occurred during the early 1970s, as evidenced by comparisons reported earlier (see Take Levels). There are also indications that the regional demand for green turtle meat in Nicaragua has not yet been satiated. For example, Miskitu Indians from the Río Coco region (border of Nicaragua and Honduras) and from the interior areas, who prior to the Sandinista/Contra war did not eat green turtle meat, have settled in Puerto Cabezas and are now consuming it (D. Castro, pers. comm.). D. Castro (pers. comm.) also reported that animals were transported by truck from Puerto Cabezas to the Río Coco region where more people are becoming accustomed to eating green turtle meat, creating a market where none previously existed. In the RAAS, it is now also trucked inland to primarily Mestizo communities that historically do not have a custom of consuming green turtle. In addition, Mestizos immigrating to the Caribbean coast from the interior or Pacific lowlands are learning to consume this inexpensive source of protein.

## Conclusion

The decrease in catch rates in Nicaragua is cause for concern for the long-term recovery of green turtle populations throughout the Caribbean that use the Nicaragua foraging grounds. Reduced catch rates observed in this study also agree with other indicators of overexploitation, such as reduced take levels (this study) and simulated negative population growth rates presented by Campbell [41], and coincides with suggestions by other studies that large juvenile and adult size classes of sea turtles are poor candidates for sustained take [102], [103], [106], [107], particularly at high levels. A more comprehensive genetic stock assessment, using longer mtDNA sequences, of the rookeries across the region, coupled with long-term monitoring, are needed to better identify distinct populations and to what degree they are impacted by or at risk from on-going turtle fisheries and unintentional take throughout the Caribbean. Furthermore, management authorities for green turtle rookeries in the region with stable or increasing nesting trends should not assume that their populations are secure given the delayed age to maturity and resulting time lag for threats on the foraging ground to manifest themselves at the nesting beach. Our results highlight the need for close monitoring of rookeries and in-water aggregations in the Caribbean. We recommend that where consumptive use still occurs, nations sharing this resource should implement scientifically based limits on exploitation to ensure sustainability and mitigate impacts to regional population diversity (see Bräutigam and Eckert [34] for recommendations).

## Supporting Information

Figure S1 **Frequencies of green turtles,** ***Chelonia mydas*** **, caught per fishing trip for landings in the (A) principal communities, (B) commercial center for the Awastara community, and (C) communities using the Refugio de Vida Silvestre Cayos Perlas fishing area.**
(PDF)

Figure S2 **Correlation in residuals ordered in time with lag time in days for the overall trend models including seasonality using landings in (A) principal green turtle,** ***Chelonia mydas*** **, fishing communities, (B) commercial center for the Awastara community, and (C) communities using the Refugio de Vida Silvestre Cayos Perlas fishing area.**
(PDF)

Figure S3 **Community specific trends in green turtle catch rates using the scaled response.** The scaled response (average turtles/day) is based on trend results shown in Figure 6 for the seven principal fishing communities in the RAAN (A) AW (Awastara), (B) DK (Dakura), and (C) SB (Sandy Bay), and in the RAAS (D) BS (Sandy Bay Sirpi), (E) RG (Río Grande Bar), (F) SN (Set Net Point), and (G) TA (Tasbapauni), assuming community specific average fishing effort in terms of nets used and trip length for the corresponding time periods. For comparison purposes, however, the period shown is from 1996 to 2011.
(PDF)

Figure S4 **Community trends in green turtle catch rates for communities fishing in the Refugio de Vida Silvestre Cayos Perlas.** Shown is estimated conditional dependence of catch rates on time by community (assuming average fishing effort in terms of nets used and trip length): (A) CB (Kahkabila), (B) HH (Haulover), (C) PL (Pearl Lagoon), and (D) SN (Set Net Point). Seasonality (E) is included in the community level model. The scaled response (average turtles/day) (F) is shown for SN only. The other communities are not shown due to the shorter time period for which data were available. Plot components are the same as in Fig. 4A and 4B. The x-axis scale corresponds to the time periods when data collection took place (starting in 1995 for SN, 1998 for CB and PL, 1999 for HH, and ending in 2011 for all communities).
(PDF)

Table S1 **Location and period of monitoring green turtle,** ***Chelonia mydas*** **, landings along the Caribbean coast of Nicaragua, 1991–2011.**
(PDF)

Table S2 **Minimum number of green turtles,** ***Chelonia mydas*** **, captured by community and in other fisheries (# of months in which data were collected) on the Caribbean coast of Nicaragua, 1991–2011.** In the RAAN, data were recorded in the communities and the commercial center of Puerto Cabezas. In some cases data were not collected at all sites for all months of the year. In those cases, two entries per cell are included, the first entry for data recorded at the community and the second entry at Puerto Cabezas (see Methods for a description of how duplication of recorded data was avoided). Total for each year includes recorded and estimated take (how the estimate was calculated is indicated by ^“f”^ or ^“k”^) when recorded data were not available for all months of the year. No data were available where cells are blank.
(PDF)

Table S3 **Summary statistics for annual catch per unit effort (CPUE) of green turtles,** ***Chelonia mydas*** **, by community and region from the Caribbean coast of Nicaragua, 1991–2011.** CPUE is based on number of turtles captured per net-day (net-day  =  number of nets set per day). No data are available where cells are blank.
(PDF)

Text S1 **Spanish translation of the abstract.**
(PDF)
