# Supplementary material for: Artisanal Green Turtle, Chelonia mydas, Fishery of Caribbean Nicaragua: I. Catch Rates and Trends, 1991–2011
Source: PLoS One. 2014 Apr 16;9(4):e94667. doi: 10.1371/journal.pone.0094667 (PMC3989241; doi:10.1371/journal.pone.0094667)

Figure S1. Frequencies of green turtles, *Chelonia mydas*, caught per fishing trip for landings in the (A) principal communities, (B) commercial center for the Awastara community, and (C) communities using the Refugio de Vida Silvestre Cayos Perlas fishing area.

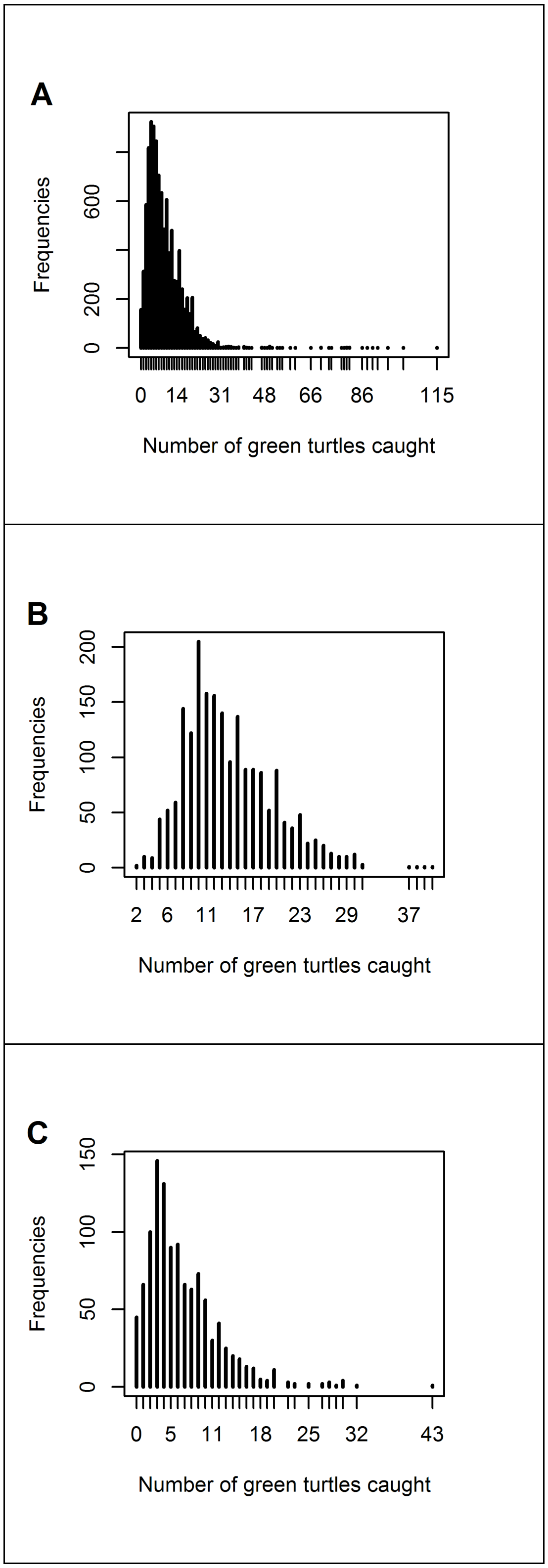

Supplement: Figure S1 — Frequencies of green turtles, Chelonia mydas , caught per fishing trip for landings in the (A) principal communities, (B) commercial center for the Awastara community, and (C) communities using the Refugio de Vida Silvestre Cayos Perlas fishing area. (PDF) [file pone.0094667.s001.pdf]
