# Supplementary material for: Artisanal Green Turtle, Chelonia mydas, Fishery of Caribbean Nicaragua: I. Catch Rates and Trends, 1991–2011
Source: PLoS One. 2014 Apr 16;9(4):e94667. doi: 10.1371/journal.pone.0094667 (PMC3989241; doi:10.1371/journal.pone.0094667)

Figure S2. Correlation in residuals ordered in time with lag time in days for the overall trend models including seasonality using landings in (A) principal green turtle, *Chelonia mydas*, fishing communities, (B) commercial center for the Awastara community, and (C) communities using the Refugio de Vida Silvestre Cayos Perlas fishing area.

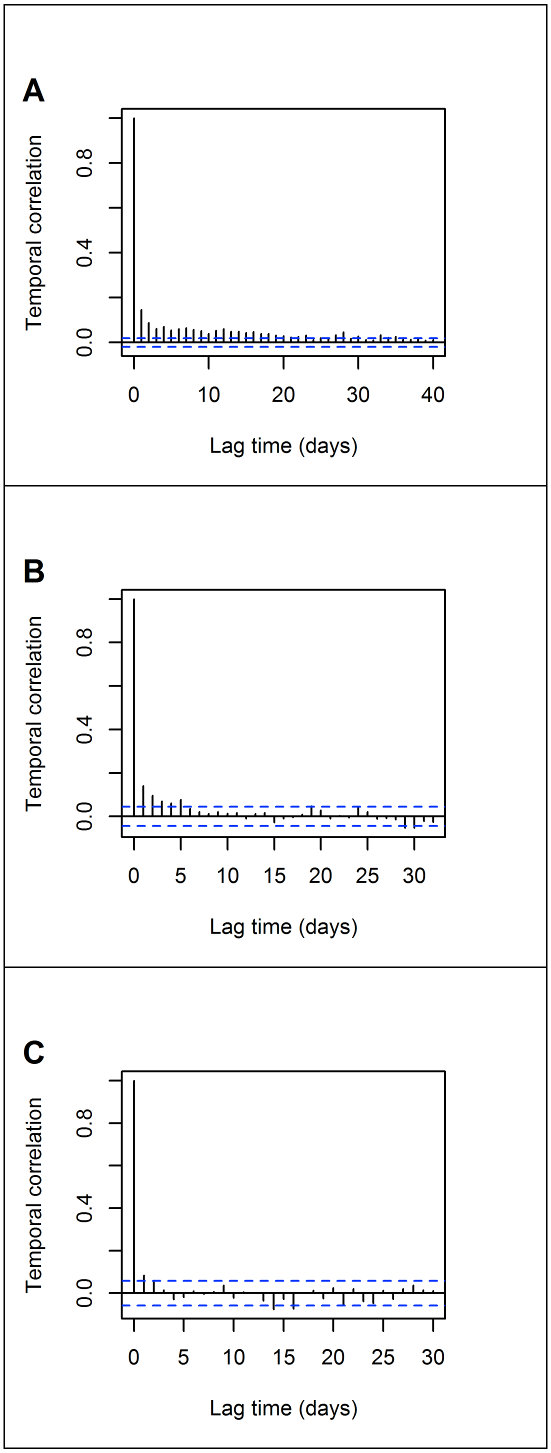

Supplement: Figure S2 — Correlation in residuals ordered in time with lag time in days for the overall trend models including seasonality using landings in (A) principal green turtle, Chelonia mydas , fishing communities, (B) commercial center for the Awastara community, and (C) communities using the Refugio de Vida Silvestre Cayos Perlas fishing area. (PDF) [file pone.0094667.s002.pdf]
