# Supplementary material for: Artisanal Green Turtle, Chelonia mydas, Fishery of Caribbean Nicaragua: I. Catch Rates and Trends, 1991–2011
Source: PLoS One. 2014 Apr 16;9(4):e94667. doi: 10.1371/journal.pone.0094667 (PMC3989241; doi:10.1371/journal.pone.0094667)

Figure S3. Community specific trends in green turtle catch rates using the scaled response. The scaled response (average turtles/day) is based on trend results shown in Figure 6 for the seven principal fishing communities in the RAAN (A) AW (Awastara), (B) DK (Dakura), and (C) SB (Sandy Bay), and in the RAAS (D) BS (Sandy Bay Sirpi), (E) RG (Río Grande Bar), (F) SN (Set Net Point), and (G) TA (Tasbapauni), assuming community specific average fishing effort in terms of nets used and trip length for the corresponding time periods. For comparison purposes, however, the period shown is from 1996 to 2011.

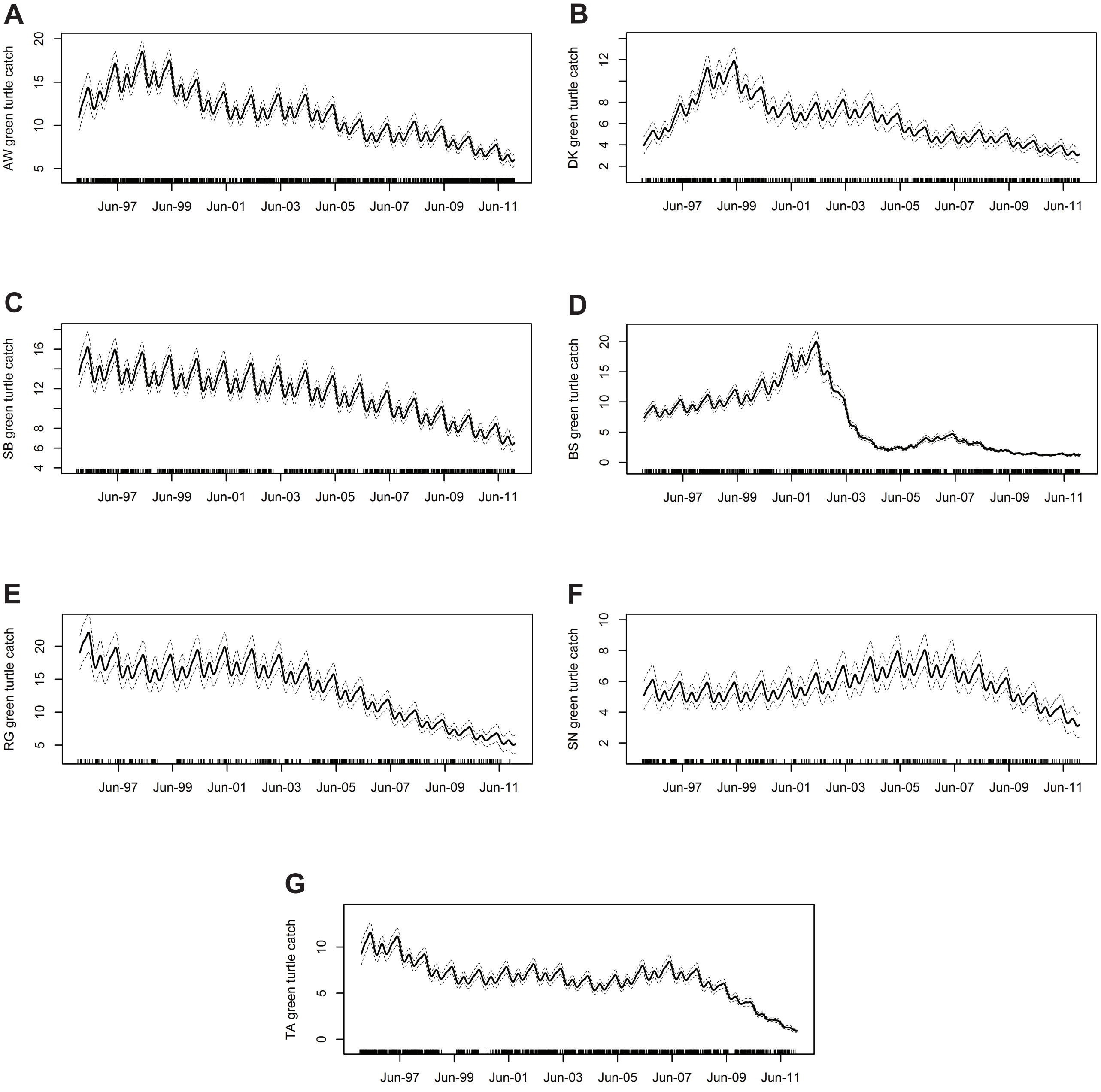

Supplement: Figure S3 — Community specific trends in green turtle catch rates using the scaled response. The scaled response (average turtles/day) is based on trend results shown in Figure 6 for the seven principal fishing communities in the RAAN (A) AW (Awastara), (B) DK (Dakura), and (C) SB (Sandy Bay), and in the RAAS (D) BS (Sandy Bay Sirpi), (E) RG (Río Grande Bar), (F) SN (Set Net Point), and (G) TA (Tasbapauni), assuming community specific average fishing effort in terms of nets used and trip length for the corresponding time periods. For comparison purposes, however, the period shown is from 1996 to 2011. (PDF) [file pone.0094667.s003.pdf]
