# Supplementary material for: Artisanal Green Turtle, Chelonia mydas, Fishery of Caribbean Nicaragua: I. Catch Rates and Trends, 1991–2011
Source: PLoS One. 2014 Apr 16;9(4):e94667. doi: 10.1371/journal.pone.0094667 (PMC3989241; doi:10.1371/journal.pone.0094667)
Supplement: Figure S4 — Community trends in green turtle catch rates for communities fishing in the Refugio de Vida Silvestre Cayos Perlas. Shown is estimated conditional dependence of catch rates on time by community (assuming average fishing effort in terms of nets used and trip length): (A) CB (Kahkabila), (B) HH (Haulover), (C) PL (Pearl Lagoon), and (D) SN (Set Net Point). Seasonality (E) is included in the community level model. The scaled response (average turtles/day) (F) is shown for SN only. The other communities are not shown due to the shorter time period for which data were available. Plot components are the same as in Fig. 4A and 4B. The x-axis scale corresponds to the time periods when data collection took place (starting in 1995 for SN, 1998 for CB and PL, 1999 for HH, and ending in 2011 for all communities). (PDF) [file pone.0094667.s004.pdf]

Figure S4. Community trends in green turtle catch rates for communities fishing in the Refugio de Vida Silvestre Cayos Perlas. Shown is estimated conditional dependence of catch rates on time by community (assuming average fishing effort in terms of nets used and trip length): (A) CB (Kahkabila), (B) HH (Haulover), (C) PL (Pearl Lagoon), and (D) SN (Set Net Point). Seasonality (E) is included in the community level model. The scaled response (average turtles/day) (F) is shown for SN only. The other communities are not shown due to the shorter time period for which data were available. Plot components are the same as in Figs. 4A and 4B. The x-axis scale corresponds to the time periods when data collection took place (starting in 1995 for SN, 1998 for CB and PL, 1999 for HH, and ending in 2011 for all communities).

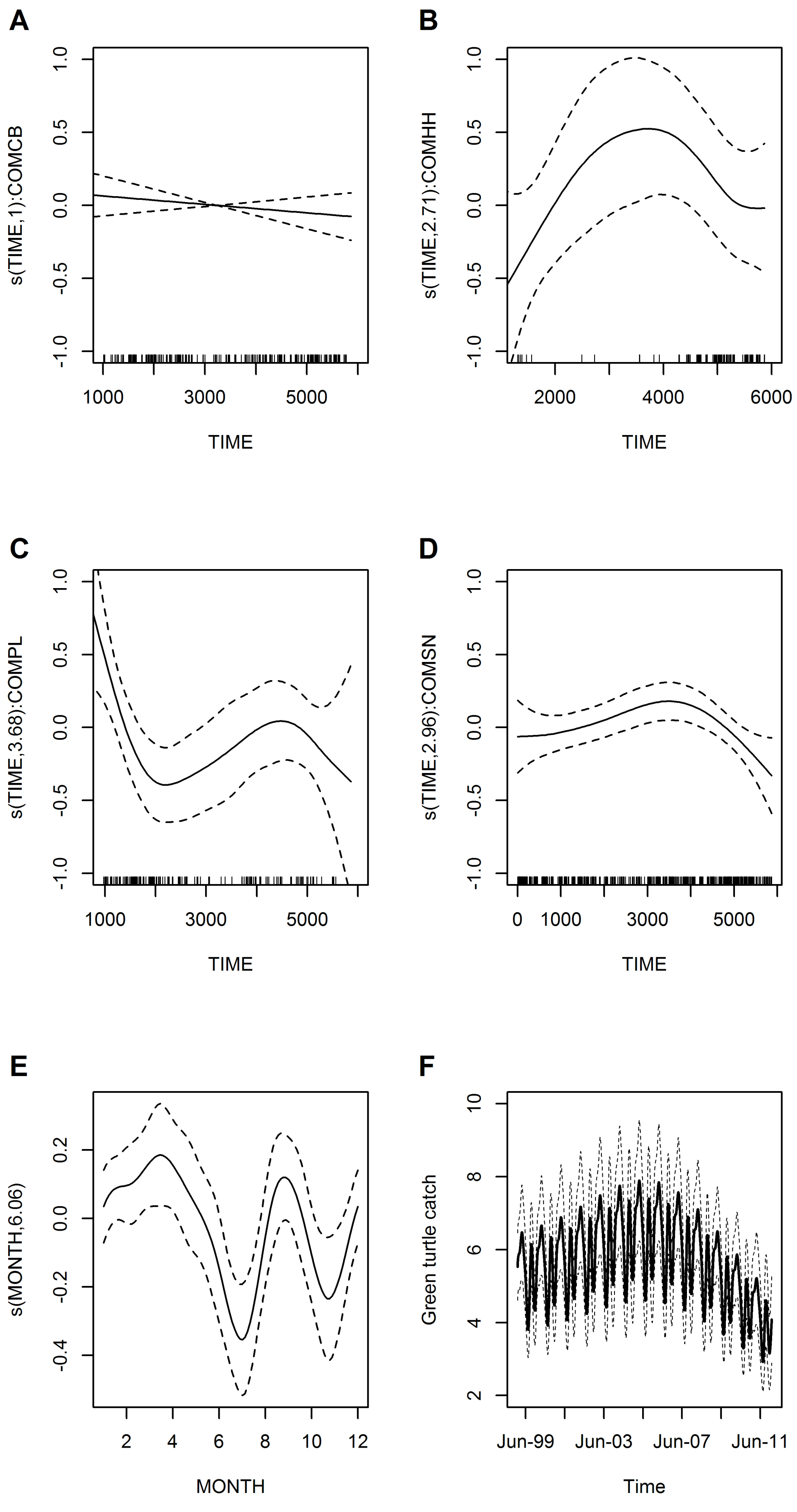
A
s(TIME,1):COMCB
TIME
B
s(TIME,2.71):COMHH
TIME
C
s(TIME,3.68):COMPL
TIME
D
s(TIME,2.96):COMSN
TIME
E
s(MONTH,6.06)
MONTH
F
Green turtle catch
Time
Jun-99
Jun-03
Jun-07
Jun-11
