# Supplementary material for: Artisanal Green Turtle, Chelonia mydas, Fishery of Caribbean Nicaragua: I. Catch Rates and Trends, 1991–2011
Source: PLoS One. 2014 Apr 16;9(4):e94667. doi: 10.1371/journal.pone.0094667 (PMC3989241; doi:10.1371/journal.pone.0094667)
Supplement: Table S1 — Location and period of monitoring green turtle, Chelonia mydas , landings along the Caribbean coast of Nicaragua, 1991–2011. (PDF) [file pone.0094667.s005.pdf]

Table S1. Location and period of monitoring green turtle, *Chelonia mydas*, landings along the Caribbean coast of Nicaragua, 1991-2011.

Site of Data Collection	Data Collection Period	% Months Data Collected (# mo)
**RAAN** (Región Autónoma Atlántico Norte)		
Awastara[a] (AW)	02/1994-01/1995 & 12/1995-12/2011	81.3 (205)
Dakura[a] (DK)	02/1994-01/1995 & 12/1995-12/2011	81.3 (205)
Puerto Cabezas[a,b,c] (Bilwi) (PC)	05-08/1991, 04/1992-03/1993, 11/1993-02/2005 & 09/2005-12/2011	90.5 (228)
Sandy Bay[a,c] (SB)	05/1992-12/1993, 02/1994-02/1995 & 12/1995-12/2011	89.7 (226)
**RAAS** (Región Autónoma Atlántico Sur)		
Awas (AS)	01/1999-12/2011	61.9 (156)
Corn Island (CI)	03/1994-08/2007	64.3 (162)
Haulover (HH)	01/1999-12/2011	61.9 (156)
Kahkabila (CB)	01/1999-12/2011	61.9 (156)
Pearl Lagoon (PL)	08/1998-12/2011	63.9 (161)
Raitipura (RP)	01/1999-12/2011	61.9 (156)
Río Grande Bar[a,d] (RG)	04, 05 & 08/1991, 01-02 & 04-05/1992, 01/1994-08/1995, 12/1995-12/2011	89.4 (220)
Sandy Bay Sirpi[a,d] (BS)	01-03/1991, 09/1991-08/1992, 10-11/1992, 01/1993-11/1994, 01/1995-12/2011	96.8 (244)
Set Net Point[a] (SN)	07/1994-12/2011	83.3 (210)
Tasbapauni[a] (TA)	11/1993-05/1994, 08/1994-12/2011	85.7 (216)

[a] Data from 1994 to April 1997 from Lagueux [18].

[b] Unpublished data for 1991 provided by C. Clark.

[c] Unpublished data from 1992 and 1993 provided by Sea Turtle Conservancy (STC, formerly Caribbean Conservation Corporation).

[d] Unpublished data from 1991 to 1993 provided by Centro de Investigaciones y Documentación de la Costa Atlántica (CIDCA).
