# Supplementary material for: Artisanal Green Turtle, Chelonia mydas, Fishery of Caribbean Nicaragua: I. Catch Rates and Trends, 1991–2011
Source: PLoS One. 2014 Apr 16;9(4):e94667. doi: 10.1371/journal.pone.0094667 (PMC3989241; doi:10.1371/journal.pone.0094667)
Supplement: Table S2 — Minimum number of green turtles, Chelonia mydas , captured by community and in other fisheries (# of months in which data were collected) on the Caribbean coast of Nicaragua, 1991–2011. In the RAAN, data were recorded in the communities and the commercial center of Puerto Cabezas. In some cases data were not collected at all sites for all months of the year. In those cases, two entries per cell are included, the first entry for data recorded at the community and the second entry at Puerto Cabezas (see Methods for a description of how duplication of recorded data was avoided). Total for each year includes recorded and estimated take (how the estimate was calculated is indicated by “f” or “k”) when recorded data were not available for all months of the year. No data were available where cells are blank. (PDF) [file pone.0094667.s006.pdf]

Table S2. Minimum number of green turtles, *Chelonia mydas*, captured by community and in other fisheries (# of months in which data were collected) on the Caribbean coast of Nicaragua, 1991-2011. In the RAAN, data were recorded in the communities and the commercial center of Puerto Cabezas. In some cases data were not collected at all sites for all months of the year. In those cases, two entries per cell are included, the first entry for data recorded at the community and the second entry at Puerto Cabezas (see Methods for a description of how duplication of recorded data was avoided). Total for each year includes recorded and estimated take (how the estimate was calculated is indicated by "[f]" or "[k]") when recorded data were not available for all months of the year. No data were available where cells are blank.

	1991[a] Recorded	Total	1992[b] Recorded	Total	1993[c] Recorded	Total	1994[d] Recorded	Total	1995[d] Recorded	Total	1996[d] Recorded	Total	1997[d] Recorded	Total
**RAAN[e]**														
Awastara					339 (5)	814[f]	292 (11)<br>3,127 (12)	3,445[f]	22 (2)<br>2,318 (12)	2,812[f]	3,417 (12)	3,417	3,787 (12)	3,787
Dakura					61 (5)	146[f]	1,022 (11)	1,080[f]	62 (2)<br>82 (12)	578[f]	370 (12)	370	696 (12)	696
Sandy Bay			1,238 (8)	1,858[f]	135 (5)<br>1,694 (12)	2,018[f]	1,455 (11)	1,570[f]	349 (3)<br>330 (12)	1,719[f]	1,607 (12)	1,607	1,876 (12)	1,876
Other communities[g]					54 (5)	130[f]	442 (12)	442	198 (12)	198	127 (12)	127	277 (12)	277
Unknown community	833 (4)	2,548[f]	1,461 (9)	1,948[f]	546 (5)	1,310[f]	0	0	0	0	0	0	325 (12)	325
Other fisheries[h]									3 (12)	3	1 (12)	1	48 (12)	48
Subtotal	833	2,548[f]	2,699	3,806[f]	2,829	4,418[f]	6,338	6,537[f]	3,364	5,310[f]	5,522	5,522	7,009	7,009
**RAAS[i]**														
Haulover														
Kahkabila														
Pearl Lagoon														
Río Grande Bar	757 (3)	3,028[f]	1,475 (4)	4,426[f]			2,253 (12)	2,253	809 (9)	1,079[f]	1,145 (12)	1,145	587 (12)	587
Sandy Bay Sirpi	377 (7)	647[f]	1,007 (10)	1,208[f]	838 (7)	1,436[f]	798 (11)	870[f]	870 (12)	870	1,146 (12)	1,146	1,488 (12)	1,488
Set Net Point							154 (6)	308[f]	406 (12)	406	226 (12)	226	292 (12)	292
Tasbapauni					336 (2)	2,016[f]	1,404 (10)	1,685[f]	2,062 (12)	2,062	2,686 (12)	2,686	2,606 (12)	2,606
Other communities[j]							171 (12)	171	30 (12)	30				
Unknown community											127 (12)	127	112 (12)	112
Other fisheries[h]														
Subtotal	1,134	3,675[f]	2,482	5,634[f]	1,174	3,452[f]	4,780	5,287[f]	4,177	4,447[f]	5,330	5,330	5,085	5,085
**Annual Total**	**1,967**	**6,223[f]**	**5,181**	**9,440[f]**	**4,003**	**7,870[f]**	**11,118**	**11,824[f]**	**7,541**	**9,757[f]**	**10,852**	**10,852**	**12,094**	**12,094**

Table S2 continued

	1998 Recorded	Total	1999 Recorded	Total	2000 Recorded	Total	2001 Recorded	Total	2002 Recorded	Total	2003 Recorded	Total	2004 Recorded	Total
**RAAN** [e]														
Awastara	4,858 (12)	4,858	4,007 (12)	4,007	2,494 (12)	2,494	2,061 (12)	2,061	2,053 (12)	2,053	2,979 (12)	2,979	3,532(12)	3,532
Dakura	801 (12)	801	754 (12)	754	408 (12)	408	306 (12)	306	532 (12)	532	477 (12)	477	465 (12)	465
Sandy Bay	1,483 (12)	1,483	1,464 (12)	1,464	1,444 (12)	1,444	978 (12)	978	821 (12)	821	856 (12)	856	1,119 (12)	1,119
Other communities[g]	247 (12)	247	81 (12)	81	4 (12)	4	58 (12)	58	64 (12)	64	95 (12)	95	34 (12)	34
Unknown community	22 (12)	22	8 (12)	8	177 (12)	177	2 (12)	2	0	0	0	0	27 (12)	27
Other fisheries[h]	14 (12)	14	1 (12)	1	5 (12)	5	7 (12)	7	1 (12)	1	2 (12)	2	7 (12)	7
Subtotal	7,425	7,425	6,315	6,315	4,532	4,532	3,412	3,412	3,471	3,471	4,409	4,409	5,184	5,184
**RAAS**[i]														
Haulover			8 (12)	8	9 (12)	9			7 (12)	7	14 (12)	14	8 (12)	8
Kahkabila	18 minimum	18	263 (12)	263	206 (12)	206	197 (12)	197	315 (12)	315	75 (12)	75	151 (12)	151
Pearl Lagoon	48 (5)	115[f]	149 (12)	149	105 (12)	105	66 (12)	66	176 (12)	176	55 (12)	55	13 (12)	13
Río Grande Bar	628 (12)	628	784 (12)	784	539 (12)	539	975 (12)	975	1,433 (12)	1,433	955 (12)	955	998 (12)	998
Sandy Bay Sirpi	1,252 (12)	1,252	597 (12)	597	982 (12)	982	623 (12)	623	1,447 (12)	1,447	674 (12)	674	359 (12)	359
Set Net Point	209 (12)	209	157 (12)	157	201 (12)	201	150 (12)	150	111 (12)	111	82 (12)	82	114 (12)	114
Tasbapauni	2,184 (12)	2,184	1,163 (12)	1,163	934 (12)	934	1,168 (12)	1,168	1,448 (12)	1,448	852 (12)	852	1,081 (12)	1,081
Other communities[j]			4 (12)	4					5 (12)	5				
Unknown community	98 (12)	98	8 (12)	8	59 (12)	59	82 (12)	82	33 (12)	33	80 (12)	80	17 (12)	17
Other fisheries[h]									7 (12)	7				
Subtotal	4,437	4,504[f]	3,133	3,133	3,035	3,035	3,261	3,261	4,982	4,982	2,787	2,787	2,741	2,741
**Annual Total**	**11,862**	**11,929**[f]	**9,448**	**9,448**	**7,567**	**7,567**	**6,673**	**6,673**	**8,453**	**8,453**	**7,196**	**7,196**	**7,925**	**7,925**

Table S2 continued

	2005		2006		2007		2008		2009		2010		2011	
	**Recorded**	**Total**	**Recorded**	**Total**	**Recorded**	**Total**	**Recorded**	**Total**	**Recorded**	**Total**	**Recorded**	**Total**	**Recorded**	**Total**
**RAAN**[e]														
Awastara	1,715 (12)<br>2,185 (6.5)	4,301[k]	2,581 (12)	2,581	1,792 (12)	1,792	1,349 (12)	1,349	2,084 (12)	2,084	2,142 (12)	2,142	2,090 (12)	2,090
Dakura	401 (12)	401	460 (12)	460	291 (12)	291	324 (12)	324	444 (12)	444	310 (12)	310	467 (12)	467
Sandy Bay	1,033 (12)	1,033	812 (12)	812	814 (12	814	1,384 (12)	1,384	1,887 (12)	1,887	1,521 (12)	1,521	1,145 (12)	1,145
Other communities[g]	58 (12)<br>22 (6.5)	102[k]												
Unknown community	0	0	0	0	0	0	0	0	0	0	0	0	1 (12)	1
Other fisheries[h]														
Subtotal	5,414	5,837[k]	3,853	3,853	2,897	2,897	3,057	3,057	4,415	4,415	3,973	3,973	3,703	3,703
**RAAS**[i]														
Haulover	7 (12)	7	18 (12)	18	10 (12)	10	140 (12)	140	316 (12)	316	185 (12)	185	691 (12)	691
Kahkabila	80 (12)	80	129 (12)	129	146 (12)	146	153 (12)	153	304 (12)	304	217 (12)	217	287 (12)	287
Pearl Lagoon	16 (12)	16	84 (12)	84	28 (12)	28	45 (12)	45	135 (12)	135	23 (12)	23	33 (12)	33
Río Grande Bar	667 (12)	667	303 (12)	303	338 (12)	338	301 (12)	301	232 (12)	232	232 (12)	232	130 (12)	130
Sandy Bay Sirpi	266 (12)	266	313 (12)	313	433 (12)	433	312 (12)	312	675 (12)	675	456 (12)	456	568 (12)	568
Set Net Point	167 (12)	167	186 (12)	186	53 (12)	53	313 (12)	313	416 (12)	416	295 (12)	295	120 (12)	120
Tasbapauni	995 (12)	995	871 (12)	871	878 (12)	878	1,010 (12)	1,010	950 (12)	950	752 (12)	752	686 (12)	686
Other communities[j]	0	0	0	0	1 (12)	1	20 (12)	20	143 (12)	143	159 (12)	159	91 (12)	91
Unknown community	42 (12)	42	119 (12)	119	28 (12)	28 (12)	2 (12)	2	0	0	0	0	0	0
Other fisheries[h]														
Subtotal	2,240	2,240[j]	2,023	2,023	1,915	1,915	2,296	2,296	3,171	3,171	2,319	2,319	2,606	2,606
**Annual Total**	**7,654**	**8,077**[k]	**5,876**	**5,876**	**4,812**	**4,812**	**5,353**	**5,353**	**7,586**	**7,586**	**6,292**	**6,292**	**6,309**	**6,309**

[a] Unpublished data for the RAAN provided by C. Clark and for the RAAS by Centro de Investigaciones y Documetación de la Costa Atlántica (CIDCA).
[b] Unpublished data for the RAAN provided by Sea Turtle Conservancy (STC, formerly Caribbean Conservation Corporation) and for the RAAS by CIDCA.
[c] Data for the RAAN provided by STC (unpublished) and Lagueux [18], and for the RAAS by CIDCA (unpublished) and Lagueux [18].
[d] Data from 1994 to April 1997 from Lagueux [18].
[e] Región Autónoma Atlántico Norte.
[f] Estimated number is based on mean number of turtles landed per month for months in which data were available for that year.
[g] Includes Krukira, Pahara, Prinzapolka, Puerto Cabezas, and/or Walpasiksa.
[h] Includes industrial shrimp trawlers, fishing boats, and/or lobster boats.
[i] Región Autónoma Atlántico Sur.
[j] Includes Awas, Bluefields, Corn Island, Kara, Karawala, Raitipura, and/or Walpa.
[k] Estimated number is based on mean number of turtles landed for the same month in the years previous and subsequent to the year for the month in which no data were available.
