# Supplementary material for: Artisanal Green Turtle, Chelonia mydas, Fishery of Caribbean Nicaragua: I. Catch Rates and Trends, 1991–2011
Source: PLoS One. 2014 Apr 16;9(4):e94667. doi: 10.1371/journal.pone.0094667 (PMC3989241; doi:10.1371/journal.pone.0094667)
Supplement: Table S3 — Summary statistics for annual catch per unit effort (CPUE) of green turtles, Chelonia mydas , by community and region from the Caribbean coast of Nicaragua, 1991–2011. CPUE is based on number of turtles captured per net-day (net-day = number of nets set per day). No data are available where cells are blank. (PDF) [file pone.0094667.s007.pdf]

Table S3. Summary statistics for annual catch per unit effort (CPUE) of green turtles, *Chelonia mydas*, by community and region from the Caribbean coast of Nicaragua, 1991-2011. CPUE is based on number of turtles captured per net-day (net-day = number of nets set per day). No data are available where cells are blank.

		1991[a]	1992[a]	1993[a]	1994[b]	1995[b]	1996[b]	1997[b]	1998	1999	2000	2001	2002	2003	2004	2005	2006	2007	2008	2009	2010	2011
**RAAN[c]**																						
AW[d]	Avg					0.119	0.126	0.150	0.151	0.147	0.141	0.106	0.112	0.130	0.096	0.108	0.084	0.077	0.084	0.092	0.064	0.062
	SD					0.065	0.086	0.090		0.094	0.115	0.100	0.068	0.108	0.065	0.076	0.058	0.053	0.057	0.064	0.041	0.031
	n					6	111	136	205	233	145	113	162	141	137	132	132	90	90	141	160	205
	Min					0.064	0.023	0.028	0.008	0.009	0.007	0.000	0.000	0.009	0.011	0.012	0.012	0.009	0.007	0.012	0.006	0.009
	Max					0.241	0.500	0.556	0.595	0.750	0.650	0.789	0.370	0.882	0.500	0.413	0.324	0.375	0.315	0.600	0.220	0.144
DK[e]	Avg					0.064	0.073	0.120	0.150	0.159	0.127	0.084	0.121	0.106	0.138	0.071	0.079	0.075	0.080	0.065	0.053	0.063
	SD					0.027	0.042	0.078	0.093	0.083	0.089	0.051	0.068	0.071	0.091	0.042	0.050	0.065	0.046	0.044	0.034	0.052
	n					6	74	107	92	90	65	52	70	61	60	65	76	55	63	80	67	82
	Min					0.039	0.011	0.029	0.033	0.023	0.026	0.000	0.027	0.014	0.014	0.016	0.013	0.017	0.021	0.017	0.013	0.009
	Max					0.100	0.229	0.500	0.450	0.400	0.500	0.250	0.450	0.357	0.417	0.259	0.267	0.400	0.200	0.278	0.178	0.354
SB[f]	Avg					0.146	0.140	0.148	0.144	0.129	0.133	0.117	0.132	0.124	0.129	0.111	0.093	0.093	0.103	0.094	0.067	0.073
	SD					0.056	0.079	0.066	0.085	0.063	0.071	0.049	0.082	0.057	0.057	0.029	0.031	0.054	0.041	0.035	0.022	0.028
	n					9	111	136	98	122	128	88	62	64	82	72	70	106	134	175	131	97
	Min					0.068	0.023	0.008	0.000	0.000	0.019	0.019	0.022	0.031	0.026	0.057	0.037	0.019	0.042	0.015	0.000	0.032
	Max					0.250	0.625	0.409	0.364	0.350	0.455	0.258	0.462	0.296	0.304	0.233	0.205	0.350	0.333	0.375	0.129	0.178
Overall	Avg					0.115	0.118	0.141	0.149	0.145	0.135	0.105	0.118	0.123	0.115	0.100	0.085	0.083	0.092	0.087	0.063	0.065
	SD					0.061	0.079	0.080	0.092	0.085	0.095	0.077	0.072	0.090	0.072	0.061	0.050	0.057	0.049	0.050	0.034	0.036
	n					21	296	379	395	445	338	253	294	266	279	269	278	251	287	396	358	384
	Min					0.039	0.011	0.008	0.000	0.000	0.007	0.000	0.000	0.009	0.011	0.012	0.012	0.009	0.007	0.012	0.000	0.009
	Max					0.250	0.625	0.556	0.595	0.750	0.650	0.789	0.462	0.882	0.500	0.413	0.324	0.400	0.333	0.600	0.220	0.354
**RAAS[g]**																						
BS[h]	Avg	0.073	0.114	0.118	0.089	0.033	0.190	0.291	0.160	0.312	0.252	0.309	0.336	0.171	0.060	0.049	0.078	0.088	0.061	0.035	0.025	0.029
	SD	0.056	0.078	0.083	0.073	0.047	0.153	0.181	0.130	0.206	0.085	0.153	0.167	0.119	0.040	0.036	0.080	0.088	0.064	0.024	0.025	0.018
	n	36	90	73	94	2	118	154	276	94	130	99	177	107	81	75	100	87	107	129	141	112
	Min	0.000	0.000	0.012	0.000	0.000	0.000	0.042	0.000	0.015	0.042	0.045	0.050	0.000	0.000	0.000	0.000	0.000	0.000	0.000	0.000	0.000
	Max	0.297	0.405	0.611	0.464	0.067	1.050	0.933	1.273	0.950	0.450	0.722	1.067	0.550	0.222	0.136	0.480	0.440	0.360	0.114	0.129	0.100
CB[i]	Avg								0.134	0.108	0.082	0.081	0.063	0.041	0.145	0.100	0.074	0.075	0.084	0.086	0.055	0.094
	SD								0.054	0.047	0.150	0.157	0.028	0.035	0.095	0.121	0.061	0.053	0.055	0.061	0.037	0.119
	n								3	14	27	37	38	11	17	13	20	27	23	35	31	29
	Min								0.074	0.030	0.000	0.011	0.019	0.000	0.017	0.024	0.000	0.000	0.020	0.000	0.000	0.025
	Max								0.178	0.217	0.806	1.000	0.167	0.125	0.333	0.417	0.217	0.208	0.222	0.333	0.156	0.667
HH[j]	Avg									0.028			0.040			0.025	0.145	0.125	0.082	0.074	0.042	0.059
	SD									0.022			0.021			0.009	0.003	0.177	0.060	0.037	0.034	0.031
	n									5			2			2	2	2	19	31	32	42
	Min									0.000			0.025			0.019	0.143	0.000	0.006	0.000	0.000	0.011
	Max									0.060			0.054			0.031	0.147	0.250	0.240	0.160	0.119	0.142
PL[k]	Avg								0.107	0.112	0.045	0.034	0.071	0.034	0.057	0.067	0.082	0.049	0.078	0.088	0.052	
	SD								0.040	0.096	0.037	0.028	0.036	0.035	0.032	0.094	0.073	0.047	0.057	0.062	0.041	
	n								11	23	33	26	20	12	3	2	17	9	7	15	6	
	Min								0.059	0.000	0.000	0.000	0.021	0.000	0.022	0.000	0.000	0.000	0.009	0.000	0.012	
	Max								0.200	0.400	0.143	0.111	0.150	0.125	0.083	0.133	0.300	0.139	0.185	0.205	0.125	

RG[l]	Avg	0.154	0.461			0.147	0.123	0.099	0.087	0.116	0.117	0.119	0.104	0.110	0.101	0.096	0.076	0.068	0.065	0.043	0.049	0.041
	SD	0.082	0.554			0.033	0.075	0.061	0.045	0.056	0.048	0.058	0.048	0.056	0.069	0.055	0.030	0.029	0.032	0.023	0.024	0.023
	n	17	23			4	36	35	37	21	35	52	65	46	53	58	31	35	30	28	27	21
	Min	0.000	0.029			0.110	0.030	0.022	0.036	0.018	0.033	0.040	0.024	0.000	0.000	0.025	0.036	0.019	0.022	0.013	0.015	0.020
	Max	0.268	2.300			0.190	0.291	0.333	0.241	0.286	0.231	0.338	0.227	0.240	0.400	0.375	0.136	0.133	0.163	0.122	0.130	0.120
SN[m]	Avg					0.256	0.213	0.177	0.201	0.215	0.233	0.194	0.233	0.242	0.283	0.265	0.286	0.210	0.277	0.203	0.183	0.145
	SD					0.150	0.161	0.086	0.137	0.139	0.185	0.214	0.106	0.170	0.255	0.191	0.199	0.106	0.190	0.152	0.157	0.118
	n					5	43	55	32	30	32	23	20	18	22	30	21	16	30	47	42	23
	Min					0.125	0.056	0.056	0.044	0.044	0.024	0.022	0.067	0.067	0.067	0.031	0.067	0.100	0.033	0.000	0.000	0.000
	Max					0.500	0.750	0.500	0.600	0.722	0.700	1.000	0.500	0.800	1.167	0.818	0.900	0.500	0.950	0.722	0.533	0.417
TA[n]	Avg					0.274	0.362	0.353	0.262	0.254	0.222	0.231	0.259	0.235	0.186	0.240	0.248	0.277	0.224	0.202	0.118	0.058
	SD					0.173	0.129	0.151	0.087	0.111	0.092	0.120	0.123	0.123	0.083	0.118	0.126	0.132	0.112	0.073	0.069	0.026
	n					10	161	165	160	68	73	176	182	161	189	162	153	144	176	160	125	85
	Min					0.173	0.133	0.094	0.028	0.031	0.063	0.022	0.000	0.038	0.050	0.020	0.063	0.063	0.042	0.048	0.027	0.022
	Max					0.750	0.909	1.000	0.733	0.667	0.500	1.000	0.818	0.889	0.700	0.600	1.000	0.667	0.607	0.417	0.316	0.175
Overall	Avg	0.099	0.184	0.118	0.089	0.223	0.263	0.284	0.188	0.233	0.194	0.207	0.242	0.187	0.149	0.168	0.167	0.175	0.152	0.121	0.076	0.056
	SD	0.075	0.291	0.083	0.073	0.157	0.165	0.171	0.125	0.170	0.125	0.154	0.159	0.128	0.113	0.136	0.139	0.143	0.130	0.104	0.085	0.061
	n	53	113	73	94	21	358	409	519	257	331	413	505	356	365	342	344	321	397	450	408	315
	Min	0.000	0.000	0.012	0.000	0.000	0.000	0.022	0.000	0.000	0.000	0.000	0.000	0.000	0.000	0.000	0.000	0.000	0.000	0.000	0.000	0.000
	Max	0.297	2.300	0.611	0.464	0.750	1.050	1.000	1.273	0.950	0.806	1.000	1.067	0.889	1.167	0.818	1.000	0.667	0.950	0.722	0.533	0.667

[a] Unpublished data provided by Centro de Investigaciones y Documetación de la Costa Atlántica (CIDCA).
[b] Data from 1994 to April 1997 from Lagueux [18].
[c] Región Autónoma Atlántico Norte.
[d] Awastara.
[e] Dakura.
[f] Sandy Bay.
[g] Región Autónoma Atlántico Sur.
[h] Sandy Bay Sirpi.
[i] Kahkabila.
[j] Haulover.
[k] Pearl Lagoon.
[l] Río Grande Bar.
[m] Set Net Point.
[n] Tasbapauni.
