# Supplementary material for: Artisanal Green Turtle, Chelonia mydas, Fishery of Caribbean Nicaragua: I. Catch Rates and Trends, 1991–2011
Source: PLoS One. 2014 Apr 16;9(4):e94667. doi: 10.1371/journal.pone.0094667 (PMC3989241; doi:10.1371/journal.pone.0094667)
Supplement: Text S1 — Spanish translation of the abstract. (PDF) [file pone.0094667.s008.pdf]

Resumen

Esta es la primera evaluación de las tasas de captura de la pesca artesanal legal de tortuga verde, *Chelonia mydas*, en la costa Caribe de Nicaragua. Los datos fueron recopilados por miembros de las comunidades, siendo monitoreados hasta 14 lugares de desembarque desde 1991 a 2011. Evaluamos los niveles de captura, y la variabilidad temporal y espacial de las tasas de captura para el total de la pesquería, por región, y por cada comunidad, usando Modelos Aditivos Generalizados Mixtos (GAMM, por sus siglas en inglés). Más de 171,556 tortugas verdes fueron sacrificadas durante el período, con un promedio mínimo estimado de 8,169 ± 2,182 sacrificadas anualmente. En conjunto, hubo un descenso estadísticamente significativo en las tasas de captura. Las tasas de captura alcanzaron picos en 1997 y 2002, seguidos de una tendencia hacia la baja, especialmente de mediados de 2008 al final del período de estudio. Tendencias hacia la baja similares fueron evidentes en ambas regiones de estudio. Las tendencias de las tasas de captura específicas de cada comunidad también indicaron descensos con caídas del 21% al 90%. El descenso en las tasas de captura en Nicaragua es motivo de preocupación a pesar de que la principal colonia de origen en Tortuguero, Costa Rica, muestra un aumento en la actividad de anidación. Las explicaciones para la aparente discrepancia entre el aumento en la tendencia de anidación en Tortuguero y la disminución de las tendencias en las tasas de captura en Nicaragua incluyen: i) un incremento del rendimiento reproductivo, ii) un período de tiempo insuficiente para observar el impacto de la pesquería sobre la colonia debido a un desfase temporal, iii) cambios en otros segmentos de la población que no hayan sido detectados debido a que sólo la actividad de anidación es monitoreada, iv) la extensión del área de alimentación en el norte de Nicaragua, la cual puede que ofrezca refugio a una porción de la colonia de Tortuguero, y/o v) una contribución de otras colonias en las áreas de pesca de tortuga en Nicaragua mayor de la esperada. Nuestros resultados destacan la necesidad de un monitoreo exhaustivo de las colonias y agregaciones en áreas de alimentación en el Caribe. Donde todavía ocurre el consumo, las naciones que comparten este recurso deberían implementar límites de explotación basados en datos científicos para asegurar la sostenibilidad y mitigar los impactos a la diversidad de la población regional.
